# Human reasoning on social interactions in ecological contexts: insights from the theory of mind brain circuits

**DOI:** 10.3389/fnins.2024.1420122

**Published:** 2024-08-01

**Authors:** Sara Isernia, Alice Pirastru, Federica Rossetto, Diego Michael Cacciatore, Marta Cazzoli, Valeria Blasi, R. Asaad Baksh, Sarah E. MacPherson, Francesca Baglio

**Affiliations:** ^1^IRCCS Fondazione Don Carlo Gnocchi Onlus, Milan, Italy; ^2^Department of Electronics, Information, and Bioengineering, Politecnico di Milano, Milan, Italy; ^3^Institute of Psychiatry, Psychology, and Neuroscience, King’s College London, London, United Kingdom; ^4^South London and Maudsley NHS Foundation Trust, London, United Kingdom; ^5^The LonDownS Consortium, London, United Kingdom; ^6^Department of Psychology, School of Philosophy, Psychology and Language Sciences, University of Edinburgh, Edinburgh, United Kingdom

**Keywords:** theory of mind, task fMRI, social cognition, mentalizing, rehabilitation

## Abstract

**Introduction:**

The relationship between neural social cognition patterns and performance on social cognition tasks in daily life is a topic of debate, with key consideration given to the extent to which theory of mind (ToM) brain circuits share properties reflecting everyday social functioning. To test the efficacy of ecological stimuli in eliciting brain activation within the ToM brain circuits, we adapted the Edinburgh Social Cognition test social scenarios, consisting of dynamic ecological contextually embedded social stimuli, to a fMRI paradigm.

**Methods:**

Forty-two adults (21 men, mean age ± SD = 34.19 years ±12.57) were enrolled and underwent an fMRI assessment which consisted of a ToM task using the Edinburgh Social Cognition test scenarios. We used the same stimuli to prompt implicit (*movie viewing*) and explicit (*silent* and *two-choice answers*) reasoning on cognitive and affective mental states. The fMRI analysis was based on the classical random effect analysis. Group inferences were complemented with supplemental analyses using overlap maps to assess inter-subject variability.

**Results:**

We found that explicit mentalizing reasoning yielded wide neural activations when two-choice answers were used. We also observed that the nature of ToM reasoning, that is, affective or cognitive, played a significant role in activating different neural circuits.

**Discussion:**

The ESCoT stimuli were particularly effective in evoking ToM core neural underpinnings and elicited executive frontal loops. Future work may employ the task in a clinical setting to investigate ToM network reorganization and plasticity.

## 1 Introduction

While navigating in the social world, individuals’ proficiency in interpreting and reacting to social cues is indispensable to allow them to behave appropriately and participate in successful social interactions (Dziobek et al., 2006; Frith, 2008; Adolphs, 2009; Henry et al., 2015; Love et al., 2015; Baez et al., 2017). This social proficiency relies on a complex set of processes known as social cognition abilities, which are acquired in infancy and continuously developed during the lifespan. Among these abilities, theory of mind (ToM) (Adolphs, 2009; Henry et al., 2013; Happé et al., 2017), the capacity to infer and respond to others’ mental states driving their behavior, assuring the prediction and adequate response to others’ social conduct (Baglio and Marchetti, 2016), is considered a key multidimensional social cognitive process, constituted by two main components, namely, affective and cognitive ToM. Specifically, reasoning on emotions refers to affective ToM, and reasoning on thoughts and beliefs refers to the cognitive ToM component.

Existing tests of ToM, however, have been criticized for their unnaturalistic and artificial nature, and therefore, the ecological validity of ToM measures to reflect social processing in daily life has gained attention (Mathersul et al., 2013). The adoption of ecological stimuli resembling the richness and complexity of daily life scenarios may reliably stimulate everyday social processing (Redcay and Moraczewski, 2020). In particular, movies depicting social interactions, rather than static images or written text, may be capable of eliciting social cognition operations in daily life. Real-world social cognitive processes rely on the online processing of dynamic multimodal, contextually embedded, temporally extended social events (Redcay and Moraczewski, 2020; Msika et al., 2024). Such processes are scarcely resembled by static and simplistic stimuli.

Some movie-based social cognition tests exist in the literature including the Movie for the Assessment of Social Cognition (MASC; Dziobek et al., 2006), the Awareness of Social Inference Test (TASIT; McDonald et al., 2003), the Awkward Moments Test (Heavey et al., 2000), and the Empathic Accuracy Paradigm (Roeyers et al., 2001). However, these tests are not without their limitations such as lacking important contextual information, offering exaggerated interactions, or being dubbed from other languages. Interestingly, virtual reality is starting to be adopted in neuropsychological assessment (Krohn et al., 2020), and the Virtual Assessment of Mentalizing Abilities (VAMA) has been proposed as an ecologically valid tool allowing evaluation of mental state reasoning in an interactive virtual environment (Canty et al., 2017). However, virtual reality systems may be not easily accessible due to the high cost related to technologies. In addition, interindividual differences in computer experience and in adaptation to the virtual environment may affect performance (Parsey and Schmitter-Edgecombe, 2013).

The Edinburgh Social Cognition test (ESCoT; Baksh et al., 2018, 2020, 2021; Poveda et al., 2022) is a measure of ToM and social norm understanding, and it has been implemented and validated in the United Kingdom and recently adapted for the Italian culture (Isernia et al., 2022a). The ESCoT assesses social abilities through everyday scenarios presented by dynamic cartoons showing a social interaction in which a character adheres to or violates a social rule in the face of a contextual request. The ESCoT provides several advantages: each cartoon measures both affective and cognitive ToM separately; its stimuli resemble the complexity of everyday social interactions; and it enables a multidimensional assessment of ToM. In fact, both social rules and contextual events are crucial to understand and infer the characters’ mental states during the social interactions in the ESCoT scenarios. Each movie depicts an expected or unexpected behavior of one character toward another based on social norms and contextual events (e.g., helping an older woman when her shopping bag breaks; not giving a pregnant woman a seat on the bus). The ESCoT scenarios show ten disparate social situations: helping the elderly, disobeying parking regulations, being considerate on the bus, cleaning up after own pet, assisting a neighbor, smoking in a prohibited area, talking in the cinema, serving a customer, skipping a bus queue, and assisting a stranger. By reasoning about the mental states of characters in those social scenarios, people are prompted to contextually embed social inferences, which are tightly dependent on how social capabilities adapt to the complexity of real-life situations.

Previous research has revealed that the ESCoT is not influenced by intellectual abilities or executive functions in healthy participants (Baksh et al., 2018; Isernia et al., 2022a), distinguishing itself from other social cognition tests (Charlton et al., 2009; Aboulafia-Brakha et al., 2011). In addition, the ESCoT has shown good diagnostic validity in discriminating between autistic and non-autistic participants (Baksh et al., 2021), individuals with and without acquired brain injuries (Poveda et al., 2022), and people with dementia with and without behavior change, unlike established tests of social cognition (Baksh et al., 2023). Given these advantages of the ESCoT, its stimuli may be a suitable way to study the neural mechanisms of mentalizing in real-life scenarios, where contextual events and social rule understanding are involved in the processing of social information.

The ToM neural-network model of Abu-Akel and Shamay-Tsoory (2011) introduces a highly complex map of interconnected neuroanatomical hubs devoted to mental state representation, attribution, and application. Based on this paradigm, mental state representations involve the temporoparietal junction (TPJ), the precuneus, the posterior cingulate cortex (PCC), and superior temporal sulcus (STS), that is, the core ToM network. In this model, partially dissociated mechanisms underlying cognitive and affective ToM have been identified in the limbic and paralimbic areas (the limbic–paralimbic ToM network): the amygdala and the ventral portion of the anterior cingulate cortex, temporal pole, and striatum are especially devoted to affective mental state understanding (affective ToM), whereas the dorsal part of the anterior cingulate cortex, temporal pole, and striatum are involved in cognitive mental state comprehension (cognitive ToM). The dissociation between affective and cognitive ToM neural hubs also involves frontal portions: the dorsomedial and dorsolateral prefrontal cortex (cognitive ToM), the ventromedial prefrontal cortex, and inferolateral and orbitofrontal cortex (affective ToM). In particular, the communication between the limbic–paralimbic ToM areas and key frontal regions enables behavior predictions based on affective and cognitive mental states.

Ecologically valid stimuli in fMRI may reveal neural regions engaged while mentalizing in a naturalistic context (Wolf et al., 2010; Redcay and Moraczewski, 2020; Hildebrandt et al., 2021). Jacoby et al. (2016) demonstrated that a non-verbal animated movie with segments eliciting emotions and beliefs was able to efficiently localize ToM functional networks, such as the bilateral STS, TPJ, precuneus, ventromedial prefrontal and dorsomedial prefrontal cortices. In addition, Pantelis et al. (2015) administered movies from a TV series in fMRI to healthy participants and autistic people to identify neural areas involved in the perception of socially awkward moments. They found activation in the right TPJ, which was selectively engaged in healthy controls and decreased in autistic people. Wolf et al. (2010) explored the neural mechanisms for both implicit and explicit mental state reasoning by adapting the MASC test into an fMRI task; they reported correspondence with typical mentalizing network areas recruited in implicit ToM reasoning during the passive movie viewing, such as the left TPJ, left precuneus, bilateral occipitotemporal cortex, and left precentral gyrus, and explicit ToM reasoning, left TPJ, left precuneus, left dorsomedial prefrontal cortex, left superior prefrontal gyrus, bilateral STS, bilateral temporal pole, and bilateral inferior frontal gyrus.

We believe that the ESCoT scenarios could be used as fMRI ecological stimuli able to elicit ToM reasoning neural patterns and differential networks for ToM affective and cognitive components.

The aim of the current study was to test the capacity of dynamic, contextually embedded, social scenarios, such as the ESCoT stimuli, in eliciting neural circuits involved in human mentalizing, and, then, propose the ESCoT scenarios as an effective and comprehensive fMRI paradigm for the assessment of cognitive and affective ToM networks.

We adapted the ESCoT into an fMRI task and administered it to a group of healthy adults. Our approach focused on verifying the engagement of ToM neural networks considering a well-recognized ToM neural-network model (Abu-Akel and Shamay-Tsoory, 2011). Specifically, when investigating the overall mentalizing reasoning compared to physical inference (control condition), we expect to find a prevalent involvement of the core ToM network, according to Wolf et al. (2010). Moreover, when the two ToM components are separately prompted, we expect to observe an extended neural pattern also involving specific cognitive and affective ToM limbic–paralimbic and executive frontal areas as detailed in the ToM neural-network model.

## 2 Methods

This is a prospective cross-sectional study conducted at the IRCCS Don Gnocchi Foundation of Milan between May 2022 and April 2023. The study protocol was approved by the “Fondazione Don Gnocchi-Milan” Ethics Committee: protocol number 08_23/02/2022. The research conformed to the ethical principles of the Helsinki Declaration revised.

### 2.1 Participants

Healthy adults were enrolled in the IRCCS Don Gnocchi Foundation clinic (Milan). They were researchers, volunteers, administrative staff, interns, and students attending the clinic. All participants enrolled agreed to take part in the research without receiving monetary compensation. They received a magnetic resonance report at the end of the study.

Before accepting eligible participants for the study, a brief clinical interview was performed to ensure they complied with the research study’s inclusion/exclusion criteria: (i) age ≥ 18; (ii) absence of neurologic and/or major psychiatric conditions; (iii) absence of pharmacological treatment with antipsychotics, antidepressants, and/or antiepileptic that may interfere with the fMRI acquisition; (iv) absence of non-corrected visual impairment able to impact the fMRI acquisition (i.e., inability to wear contact lenses for myopia); (v) absence of hearing impairment able to impact the behavioral assessment; (vi) absence of MRI contraindications (i.e., pacemaker, metal implants, and crystalline surgery in the last month). All participants read and signed the written informed consent.

We enrolled a total of 42 healthy adults (21 men, mean age ± SD = 34.19 years ±12.57, mean full-time years of education ± SD = 16.27 years ±3.08). Among these, seven participants were excluded from the analyses: one due to a brain lesion and six due to low-quality MRI data due to movement artifacts (head motion above 2 mm/2°).

### 2.2 Procedure

After recruitment into the research study, participants were involved in an individual session in the clinic to perform: (i) an MRI acquisition lasting approximately 40 min in total and (ii) a neuropsychological assessment lasting approximately 1 h. The MRI examination included brain structural MRI sequences to study brain morphometry and exclude gross brain abnormalities, and the ESCoT fMRI ToM task, preceded by 10 min of familiarization with the task instructions and stimuli outside the MRI scanner (see Section 2.3.2). The neuropsychological assessment comprised a test battery to evaluate both non-social cognitive level and social cognitive abilities (see Section 2.3.3).

### 2.3 Materials

#### 2.3.1 fMRI ToM task implementation

**Figure 1:**
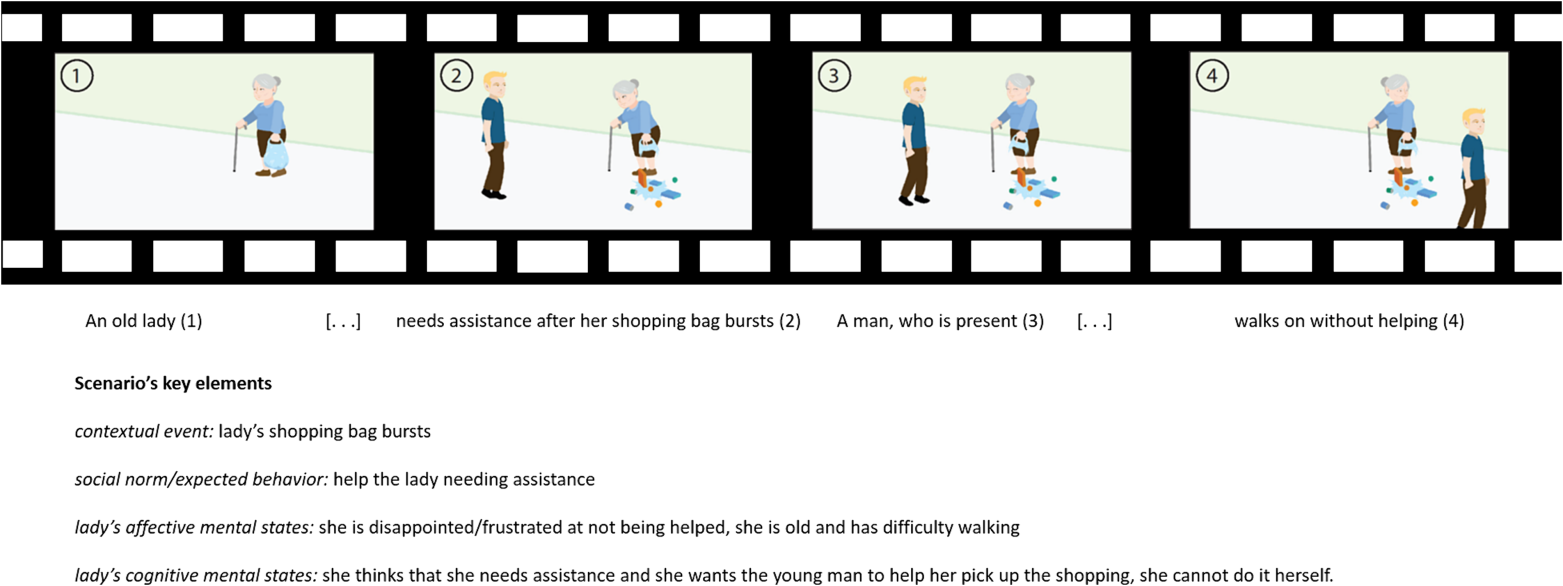
Storyboard of an ESCoT movie (“Helping the elderly”) and the description of the social scenario of the movie.

A new fMRI ToM task was derived from the Edinburgh Social Cognition Test (ESCoT, Baksh et al., 2018), originally developed in the United Kingdom (Baksh et al., 2018, 2020, 2021; Poveda et al., 2022) and then adapted for the Italian language (Isernia et al., 2022a), assessing affective and cognitive ToM and social norm understanding with an ecological and multidimensional approach. The test consists of 11 cartoon-style silent animations (30 s each) depicting real-life social interactions. The animations show social interactions complying with or violating social norms in a daily life context (e.g., assisting a stranger, skipping a bus queue, and smoking in a prohibited area). Each animation evaluates separately cognitive and affective components of ToM, and interpersonal and intrapersonal comprehension of social norms (i.e., social knowledge). In its original version, participants are invited to watch animations and then answer open-ended questions about what happened in the videos (animation comprehension), what a character is thinking (cognitive ToM), how a character is feeling (affective ToM), whether a character behaves as other people should behave (interpersonal comprehension of the social norm), and whether the participant would have acted the same as the character (intrapersonal comprehension of the social norm). Each answer is scored from 0 to 3 points, with 3 points indicating an optimal interpretation of the social dynamics, explicitly extracting and integrating relevant social information and contextual factors influencing characters’ behavior. A score of 2 indicates a response that explicitly extracts the relevant social information but does not integrate it into the context related to the interaction or refers to the extraction of low-level social information related to the context. A score of 1 recognizes a non-social information response, even with the mention of contextual requests. A score of 0 indicates an “I do not know” answer. For a detailed description of the scoring procedure, please refer to Isernia et al. (2022a) and Baksh et al. (2018) (see Figure 1 for an example).

For the fMRI adaptation, the ESCoT animation stimuli were adapted to have the same duration (23 s), specifically the vignettes have been shortened by cutting only the start or the end frames when the social interaction has not yet occurred/was already concluded. In addition, instructions for both open- (silent) and closed-ended questions were implemented for each animation assessing social cognition. Then, before implementing the definitive version of the task, pilot versions were administered inside the MRI scanner. The first pilot version of the task included both questions on ToM and comprehension of social norms. However, after preliminary analyses reporting inconsistent neural activations related to social norm comprehension among participants, the task was modified to assess only ToM (affective and cognitive components).

**Figure 2:**
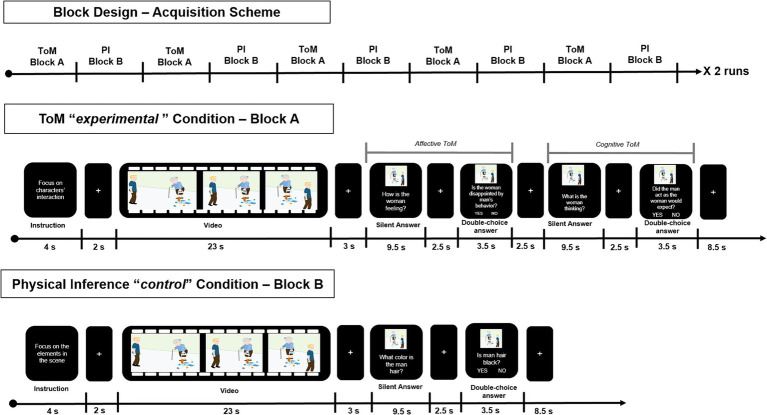
Overview of the block design of the task-fMRI experiment. The items of the two blocks (namely, Block A—Theory of Mind *Experimental* Condition, and Block B—Physical Inference *Control* Condition) are shown, together with the stimuli duration, according to the order of presentation.

In its final version, the ESCoT fMRI task has been implemented in a way that the same stimuli (video clips) were used to investigate neural correlates elicited by *ToM reasoning* (experimental condition) and *physical inference* (control condition). To this purpose, the ESCoT fMRI task consisted of two blocks (A–B) modeling two different conditions: the ToM *experimental* condition (A) and the physical inference (PI) *control* condition (B). Moreover, in each block, the same stimuli were used to test the neural activations of *implicit reasoning*, namely, the reasoning spontaneously elicited by the stimulus itself, not resulting from a specific question, and the *explicit reasoning*, which instead consists of reasoning elicited by questions that purposely direct attention toward mental states or physical elements (see Figure 2).

Specifically, the ToM experimental block (A) included the following items:

1. *Social cognition instructions*: written instructions for the movie scene (*“Focus on characters’ interactions”*) lasting on the screen for 4 s;
2. *Implicit ToM reasoning*: cartoon-style animation movie watching, lasting 23 s;
3. *Explicit affective ToM reasoning – Silent answer*: silent answer to affective ToM question (*“How is the woman feeling?”*), lasting on the screen for 9.5 s;
4. *Explicit affective ToM reasoning – Closed-ended answer*: two -choice answer to affective ToM question (*“Is the woman disappointed by the man behavior?”*), lasting on the screen for 3.5 s;
5. *Explicit cognitive ToM reasoning – Silent answer*: Silent answer to cognitive ToM question (*“What is the woman thinking?”*), lasting on the screen for 9.5 s;
6. *Explicit cognitive ToM reasoning – Closed-ended answer*: two-choice answer to cognitive ToM question (*“Did the man act as the woman would expect?”*), lasting on the screen for 3.5 s.

The PI *control* block (B) included the following items:

1. *Physical instructions:* written instruction for the movie scene (“*Focus on the elements in the scene*”) lasting 4 s;
2. *Implicit physical inference:* cartoon-style animation movie watching, lasting 23 s;
3. *Explicit physical inference – Silent answer:* silent answer to PI question (“*What color is the man’s hair?*”), lasting on the screen for 9.5 s;
4. *Explicit physical inference – Closed-ended answer:* two-choice answer to PI question (“*Is the man’s hair black?*”), lasting on the screen for 3.5 s.

All items presented in each block were interleaved by a white fixation cross on a black background of variable duration (ranging between 2 to 3 s, and 6 s between blocks) (see Figure 2).

The fMRI task included a total of 10 animation movies (the remaining animation was used for familiarization of the task outside the MRI scanner), each of which was administered twice: once during the ToM *experimental* condition and once for the PI *control* condition, according to a randomized order. The task administration was split into two separate, but sequential runs (5 movies each) during the same scanning session. Both the task sessions lasted approximately 10 min for a total fMRI task duration of 20 min. The closed-ended two-choice answer was recorded using a dedicated device, namely, an Evoke Response Pad System (Resonance Technology Inc.), and consisted of pressing a button with the index or middle finger to indicate positive or negative answers, respectively. The task was implemented and successively administered using E-Prime 3.0 (Psychology software tools)^1^.

#### 2.3.2 MRI data acquisition

The data were acquired on a 3 T Siemens Prisma scanner (Erlangen, Germany) equipped with a 64-channel head/neck coil. The acquisition protocol included the following: (1) a T1-3D magnetization prepared rapid acquisition with gradient-echo (MPRAGE) sequence with a repetition time (TR) = 2,300 ms, echo time (TE) = 3.1 ms, isotropic resolution = 0.8 × 0.8 × 0.8 mm^3^, 224 slices, which was used as an anatomical reference; (2) a sagittal fluid-attenuated inversion recovery (FLAIR) sequence was also acquired TR = 5,000 ms, TE = 394 ms, resolution = 0.8 × 0.8 × 1 mm^3^, acquisition matrix = 288 × 320, 176 slices, to exclude gross brain abnormalities; (3) an accelerated GE sequence with TR = 2000 ms, TE = 30 ms, resolution 3 × 3 × 3 mm^3^, multi-slice acceleration factor = 2, 52 slices, 333 measurements, 2 runs for fMRI.

The ESCoT visual stimuli were delivered using E-Prime 3.0 [Psychology software tools (see Footnote 1)] by means of a NordicNeuroLab system^2^ comprising an “in-room viewing device.” Specifically, an MR-compatible display was located at the end of the gantry, and a mirror was placed on the head coil to allow the participant to see the monitor. The stimuli administration was synchronized with the MR acquisition by means of a dedicated device (SyncBox). The participants were trained before entering the MRI scanner and performed a trial mimicking the fMRI experiment structure involving an ESCoT test video.

#### 2.3.3 Neuropsychological assessment

The neuropsychological test battery included both conventional non-social cognitive measures and social cognition tools.

Non-social cognitive measures comprised the following:

*Montreal Cognitive Assessment* (MoCA; Conti et al., 2015; Santangelo et al., 2015) to assess global cognition. The total score ranging from 0 to 30 (greater cognitive level) was adjusted for age and years of education based on the instructions of Santangelo et al. (2015).

*Trail Making Test* (TMT; Giovagnoli et al., 1996) to evaluate shifting. Performance time was recorded both for TMT parts A and B, and total scores were adjusted for age and years of education according to Giovagnoli et al. (1996).

*Stroop Color-Word Test* (Strauss et al., 2006) to assess inhibition. Performance time and errors were registered, and total time and total errors were computed and adjusted for age and years of education according to Caffarra et al. (2002).

*Digit Span Test* (Monaco et al., 2013) to assess short-term and working memory. Forward and backward total scores were computed and adjusted for sex, age, and years of education based on the instructions of Monaco et al. (2013).

*Symbol Digit Modality Test* (SDMT; Sheridan et al., 2006) to evaluate processing speed and attention. The total score was computed and adjusted according to Rao (1990).

Social cognition measures included the following:

*Yoni Task* (Shamay-Tsoory and Aharon-Peretz, 2007; Isernia et al., 2022c) to evaluate cognitive and affective, first-order and second-order ToM with visual cartoon-like stimuli. The 48-item version was administered, and total accuracy and total response time indexes (range 0–1) were computed and adjusted for sex, age, and education according to Isernia et al. (2022c).

*Reading the Mind in the Eyes test* (RME, Baron-Cohen et al., 2001) to evaluate ToM through photographs of the eye region expressing complex mental states. The total score (range 0–36) was computed and adjusted according to Maddaluno et al. (2022).

### 2.4 MRI data analysis

#### 2.4.1 fMRI data preprocessing

The EPI functional data were preprocessed, according to a standard pipeline, using the Statistical Parametric Mapping toolbox (SPM12)^3^ running on MATLAB (MathWorks)^4^. The first 10 acquired volumes were considered as ‘dummy scans’ and were discarded to account for magnetization to reach the steady state. The two runs were preprocessed together by setting two different sessions. The first step of the preprocessing involved motion correction and realignment of functional volumes to an average reference volume. The degree of head motion was assessed, and participants with movements exceeding the threshold set at 2 mm/2° were excluded from further analysis. Then, the co-registration with individuals’ anatomical volumes (MPRAGE) was performed. Specifically, the MPRAGE anatomical volumes were preprocessed using the FMRIB Software Library v6.0 (FSL)^5^ according to the following steps: bias field correction (Tustison et al., 2010) and brain extraction (Smith, 2002; Jenkinson et al., 2005). The individual volumes were successively used as anatomical references for the registration of functional volumes, performed in SPM, at the subject level. The last steps involved segmentation and normalization to the standard MNI template and smoothing (8 mm full width at half-maximum isotropic Gaussian). The preprocessed volumes served as input to the following first-level statistical analyses.

#### 2.4.2 fMRI statistics

*A priori* sample size calculation: in accordance with the recommendations reported in Szucs and Ioannidis (2020), we used G*Power (version 3.1.9.7) to *a priori* estimate the sample size for the study. A total of 31 participants resulted as necessary to achieve a power equal to 0.85 for one-sample *t*-test analysis (d = 0.5) and α threshold = 0.05. We considered enrolling an additional 15% of subjects to account for eventual exclusions from fMRI analysis due to low-quality data or due to non-compliance with the MRI scan.

##### 2.4.2.1 First-level analyses

The general linear model (GLM) was used to construct and fit the statistical model on the BOLD response to perform the first-level analysis at the subject level. Every item was modeled as a single event inside each block (ToM “experimental” block and PI “control” block), namely, movie viewing, silent answering, and two-choice question answering, and represented the regressors of interest. The six motion parameters were instead inserted in the model as nuisance regressors. Seven different contrasts were considered comparing the different items between the two conditions (i.e., ToM Experimental condition and PI Control condition), specifically, (1) implicit ToM reasoning vs. implicit PI (movie viewing following “ToM instruction” vs. movie viewing following “PI instruction”), (2) explicit ToM reasoning vs. explicit PI silent answer, (3) explicit ToM reasoning vs. explicit PI closed-ended answer, (4) explicit affective ToM (aToM) reasoning vs. explicit PI silent answer, (5) explicit aToM reasoning vs. explicit PI closed-ended answer, (6) explicit cognitive ToM (cToM) reasoning vs. explicit PI silent answer, and (7) explicit cToM reasoning vs. explicit PI closed-ended answer.

To directly test the selective activation of the affective and cognitive ToM dimension, four additional contrasts, directly testing the differences between ToM components during both silent and closed-ended explicit reasoning, have been computed: (8) explicit aToM reasoning vs. explicit cToM reasoning silent answer, (9) explicit cToM reasoning vs. explicit aToM reasoning silent answer, (10) explicit aToM reasoning vs. explicit cToM reasoning closed-ended answer, and (11) explicit cToM reasoning vs. explicit aToM reasoning closed-ended answer.

Moreover, to complement the classical GLM factorial analysis, subjects’ interindividual variability was performed. Specifically, subject-specific activation maps derived for all the above-mentioned contrasts have been used to compute threshold-dependent overlap maps representing the proportion of subject activation in a given region of interest (ROI) (Seghier and Price, 2016). The ROIs were defined according to an in-house developed atlas-derived inclusive mask previously described in Isernia et al. (2022b), comprising the cerebral areas relevant to ToM reasoning according to Abu-Akel and Shamay-Tsoory’s model (Abu-Akel and Shamay-Tsoory, 2011). The subject-specific activation maps were derived according to the following thresholds: p_unc_ < 0.001 and cluster size ≥30.

##### 2.4.2.2 Second-level analyses

The resulting subject-level contrasts were used to perform second-level group analysis, modeled in the GLM as one-sample *t*-tests. The statistics were restricted using an in-house developed atlas-derived inclusive mask previously described in Isernia et al. (2022b), to the cerebral areas relevant to ToM processing according to the Abu-Akel and Shamay-Tsoory’s model (Abu-Akel and Shamay-Tsoory, 2011). In brief, the mask was composed of 11 bilateral non-overlapping ROIs, constituting the four ToM circuits depicted by the model: the ‘*Core ToM Network’*, composed of TPJ, precuneus, and PCC and the anterior division of the STS; the ‘*Limbic–Paralimbic ToM Network’*, composed by anterior cingulate cortex (ACC), TP, dorsal striatum, ventral striatum, and amygdala; the ‘*Cognitive Execution Loop’*, composed by dorsal medial and dorsal lateral PFC; the ‘*Affective Execution Loop’*, composed by the orbitofrontal cortex and ventromedial PFC, and the inferolateral PFC. The functional activation maps were considered statistically significant for p_FWE_ < 0.05 considering the family-wise error (FWE) correction for multiple comparisons to account for false positives. A threshold on cluster size was also set to consider clusters larger than 30 voxels.

## 3 Results

### 3.1 Participants

Thirty-five participants (18 men, mean age ± SD = 34.23 years ±12.72, mean years of education ± SD = 16.46 years ±2.70) were included in the analyses. Table 1 reports the cognitive and psychosocial characteristics of the participants included in the analyses. Participants showed high performance on all the neuropsychological tests.

**Table 1:** Neurocognitive profile of the sample.

Domain	Measure	M, SD (95% CI)	Cut-off
Global cognitive level	MoCA	24.90, 2.84 (23.96–25.84)	<15.50
Shifting	TMT		
	Part A	35.80, 13.10 (31.46–40.14)	>94
	Part B	109.00, 39.30 (95.98–122.02)	>283
Inhibition	Stroop		
	Time	21.50, 5.78 (19.58–23.41)	>36.92
	Errors	0.57, 1.18 (0.18–0.96)	>4.24
Short-term memory	Digit span Forward	6.18, 1.52 (5.68–6.68)	<4.26
Working memory	Digit span Backward	4.85, 1.68 (4.29–5.41)	<2.65
Processing speed	SDMT	55.60, 12.00 (51.62–59.58)	<37.90
ToM	Yoni task		
	Total accuracy	0.91, 0.08 (0.88–0.94)	-
	Affective	19.00, 2.05 (18.32–19.68)	
	Cognitive	19.20, 1.86 (18.58–19.82)	
	First-order	15.80, 0.29 (15.70–15.90)	
	Second-order	22.30, 3.32 (21.20–23.40)	
	Total response time	0.92, 0.03 (0.91–0.93)	
	RME	28.10, 2.58 (27.24–28.95)	<19.24

CI, confidence interval; M, mean; RME, Reading the Mind in the Eyes test; SD, standard deviation; SDMT, Symbol Digit Modality Test; ToM, theory of mind; TMT, Trail Making Test, MoCA, Montreal Cognitive Assessment Test.

### 3.2 fMRI task performance

The task-fMRI performance for the closed-ended questions, for both the ToM experimental and PI control conditions, is reported in Table 2. Specifically, the reaction times (RT), namely, the elapsed time between the question presentation and the participants pressing the button, the number of missing answers, and the number of wrong answers were assessed for the affective ToM (aToM), cognitive ToM (cToM), and PI closed-ended questions. All participants correctly answered at least 75% of overall questions with a low rate of missing/wrong answers and average RT below the 3,000-ms time limit.

**Table 2:** Performance indexes of closed-ended questions recorded during MRI examination.

Domain	Reaction Times [ms] M, SD (95% CI)	# Missing Answer M, SD (95% CI)	# Wrong Answer M, SD (95% CI)
AToM	2178.2, 292.9 (2081.1–2275.2)	0.4, 0.6 (0.2–0.6)	1.1, 0.6 (0.9–1.3)
CToM	2342.6, 259.6 (2256.6–2428.6)	1.4, 1.1 (1–1.7)	0.3, 0.5 (0.2–0.5)
PI	1731, 239.5 (1651.6–1810.3)	0.2, 0.5 (0.1–0.4)	0.3, 0.5 (0.2–0.5)

ms, milliseconds; M, mean; SD, standard deviation; aToM, affective theory of mind; cToM, cognitive theory of mind; PI, physical inference.

### 3.3 fMRI GLM results

**Figure 3:**
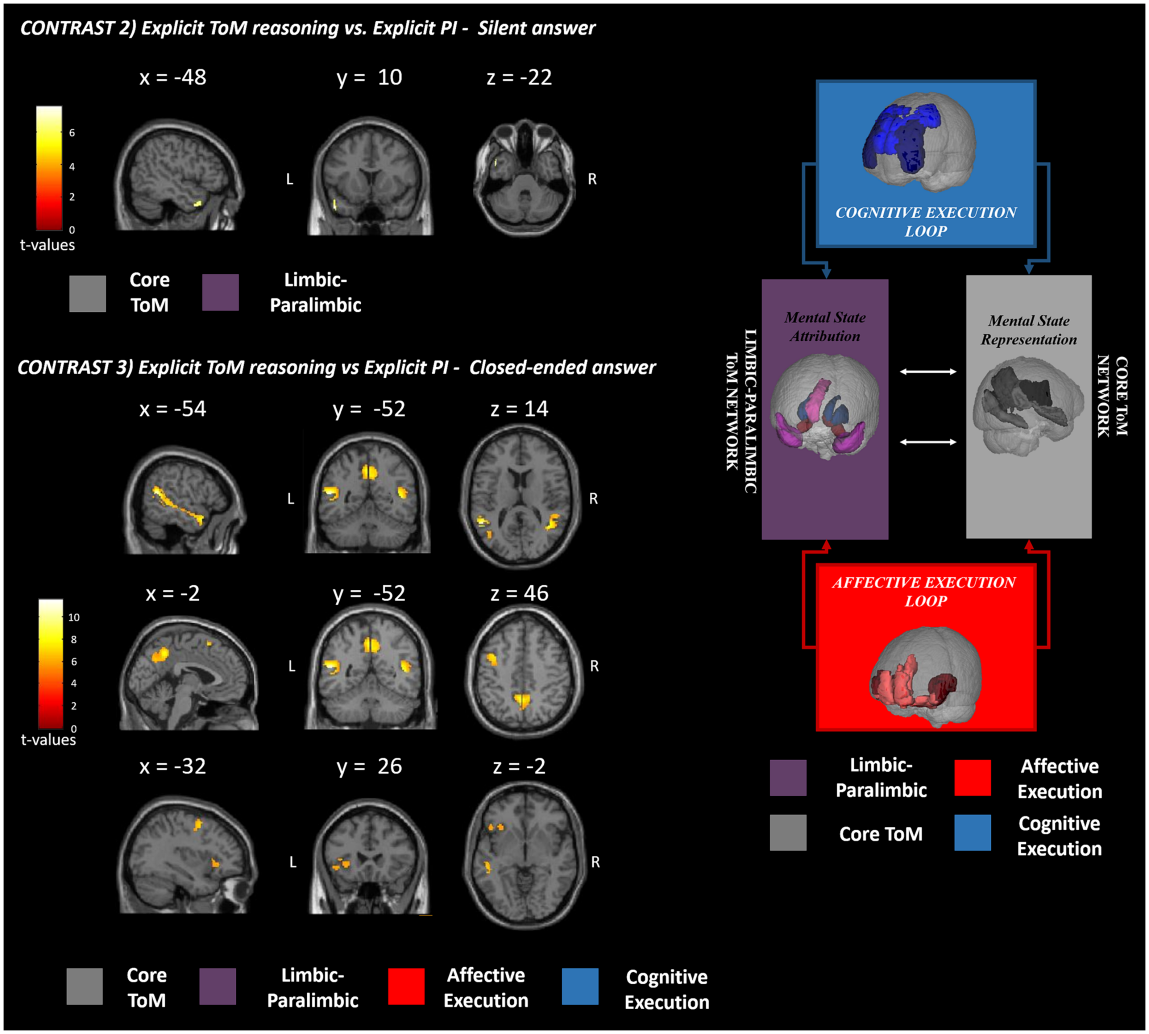
fMRI GLM results. This figure shows the fMRI results observed in the explicit (contrasts 2 and 3) ToM reasoning with respect to the physical inference control condition. The significant clusters of activation are reported in red-yellow expressing the t-values according to the reported color bar. ToM, theory of mind; PI, physical inference; L, left; R, right. The significant clusters for each contrast are mapped according to the Abu-Akel and Shamay-Tsoory ToM model depicted on the right [adapted from Isernia et al. (2022b)].

The results of the fMRI analysis are reported in detail in Table 3 and Figure 1 for the ToM performance vs. PI and separately for affective ToM and cognitive ToM vs. PI in Table 4 and Figure 2. Specifically, for the first contrast (*implicit ToM reasoning* vs. *implicit PI*), investigating the implicit ToM reasoning, no significant neural activation was retrieved. For the second contrast (*explicit ToM reasoning* vs. *explicit PI silent answer*), the functional activations were located in the left TP, both superior and middle (BA 21, 22, 38) (Figure 3). The third contrast (*explicit ToM reasoning* vs. *explicit PI closed-ended answer*) yielded significant activation in the bilateral temporal cortex, specifically superior and middle temporal gyri (BA 21, 22, 39), and precuneus (BA 7), left temporal pole (BA 38), inferior frontal cortex (pars orbitalis and pars triangularis), and insula (Figure 3). Furthermore, significant clusters were retrieved in the left middle frontal cortex and precentral gyrus.

**Table 3:** MRI GLM Results.

		MNI Coord [x, y, z] mm	Anatomical Region (AAL)	BA	p_FWE_	Cluster size	T
**Explicit ToM reasoning** ***vs.*** **Explicit PI - SA** **(Contrast 2)**	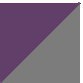	**[-50; 10; -34]**	**L Temporal Pole/ Middle TP**	**21**	**<0.001**	**68**	**7.60**
		[-54 8 -20]	L Middle TP	22			
		[-52 12 -24]	L Superior TP	38			
**Explicit ToM reasoning** ***vs.*** **Explicit PI - CA** **(Contrast 3)**	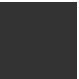	**[-54 -52 14]**	**L Middle Temporal C**	-	**<0.001**	**602**	**11.52**
		[-52 -30 2]	L Superior Temporal G	21			
		[-50 -33 2]	L Middle Temporal G	22			
	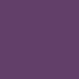	**[-50 12 -24]**	**L Temporal Pole**/Superior TP	**38/21**	**<0.001**	**235**	**9.47**
	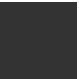	**[42 -56 14]**	**R Temporal Middle**	-	**<0.001**	**317**	**8.27**
		[42 -58 17]	R Superior Temporal G	39			
	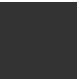	**[-2 -52 46]**	**Parietal Lobe/L Precuneus**	**7**	**<0.001**	**281**	**8.48**
	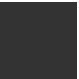	**[2 -52 46]**	**R Precuneus**	**-**	**<0.001**	**318**	**7.86**
		[2 -62 34]	R Precuneus	7			
	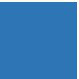	**[-40 8 48]**	**L Middle Frontal Gyrus/L Precentral C**	**-**	**<0.001**	**295**	**7.41**
	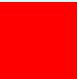	**[-32 26 -2]**	**L insula**	**-**	**<0.001**	**84**	**6.18**
		[-40 22 8]	L Frontal L Inferior Triangular C	13			
	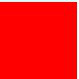	**[-46 26 -6]**	**L Frontal Inferior Orbital C**	**-**	**0.001**	**37**	**6**

AAL, automatic anatomical labeling atlas; BA, Brodmann areas; FWE, family-wise error correction for multiple comparisons; MNI, Montreal Neurological Institute standard; L, left; R, right; ToM, theory of mind; PI, physical inference; aToM, affective theory of mind; cToM, cognitive theory of mind; SA, silent answering; CA, closed-ended answering; C, cortex; G, gyrus. The information relative to cluster peaks is reported in bold.

**Table 4:** MRI GLM Results.

		MNI Coord [x, y, z] mm	Anatomical Region (AAL)	BA	p_FWE_	Cluster size	T
**Explicit aToM reasoning** ***vs.*** **Explicit PI - CA (Contrast 5)**	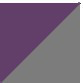	**[-52 12 -22]**	**L Temporal Pole**/ Superior TP	**38**	**<0.001**	**1050**	**13.09**
		[-54 -52 14]	L Middle Temporal C	-			
		[-50 14 -32]	L Middle TP	-			
	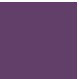	**[56 8 -18]**	**R Middle TP/Middle Temporal G**	**21**	**<0.001**	**84**	**7.08**
		[52 18 -24]	R Superior TP/Superior Temporal G	-			
	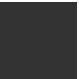	**[-4 -66 38]**	**L Precuneus**	**7**	**<0.001**	**209**	**7.4**
	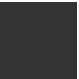	**[4 -62 36]**	**R Precuneus**	**7**	**<0.001**	**265**	**6.84**
	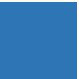	**[-40 6 46]**	**L Middle Frontal G/Precentral G**	**-**	**<0.001**	**383**	**11.09**
	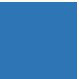	**[-6 14 58]**	**L Superior Frontal G/Supp. Motor Area**	**-**	**<0.001**	**58**	**9.51**
	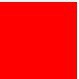	**[-46 26 -6]**	**L Inferior Frontal G/Orbital G**	**-**	**<0.001**	**291**	**8.35**
		[-32 26 0]	L Insula	-			
	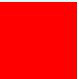	**[-46 18 16]**	**L Inferior Frontal G/Triangular G**	**-**	**<0.001**	**70**	**8**
		[-36 26 2]	L Triangular G	47			
**Explicit cToM reasoning** ***vs.*** **Explicit PI - SA** **(Contrast 6)**	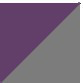	**[-50 10 -34]**	**L Middle TP/Middle Temporal G**	**21**	**<0.001**	**113**	**8**
		[-52 12 -24]	L Superior TP/Superior Temporal G	38			
**Explicit cToM reasoning** ***vs.*** **Explicit PI - CA** **(Contrast 7)**	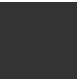	**[-54 -54 16]**	**L Temporal Middle/Superior Temporal G**	**-**	**<0.001**	**212**	**7.2**
		[-44 -66 15]	L Temporal Middle/Middle Temporal G	39			
	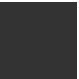	**[46 -52 14]**	**R Temporal Middle/Superior Temporal G**	**-**	**<0.001**	**397**	**7.46**
		[42 -58 18]	R Temporal Middle/Superior Temporal G	39			
	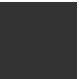	**[-2 -52 46]**	**L Precuneus**	**7**	**<0.001**	**127**	**7.46**
	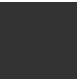	**[2 -50 48]**	**R Precuneus**	**7**	**<0.001**	**182**	**7.38**

AAL, automatic anatomical labeling atlas; BA, Brodmann areas; FWE, family-wise error correction for multiple comparisons; MNI, Montreal Neurological Institute standard; L, left; R, right; ToM, theory of mind; PI, physical inference; aToM, affective theory of mind; cToM, cognitive theory of mind; SA, silent answering; CA, closed-ended answering; C, cortex; G, gyrus. The information relative to cluster peaks is reported in bold.

Explicit ToM reasoning was also investigated separately for the affective (contrasts 4 and 5) and cognitive (contrasts 6 and 7) ToM components through the use of silent open-ended questions and two-choice closed-ended questions.

**Figure 4:**
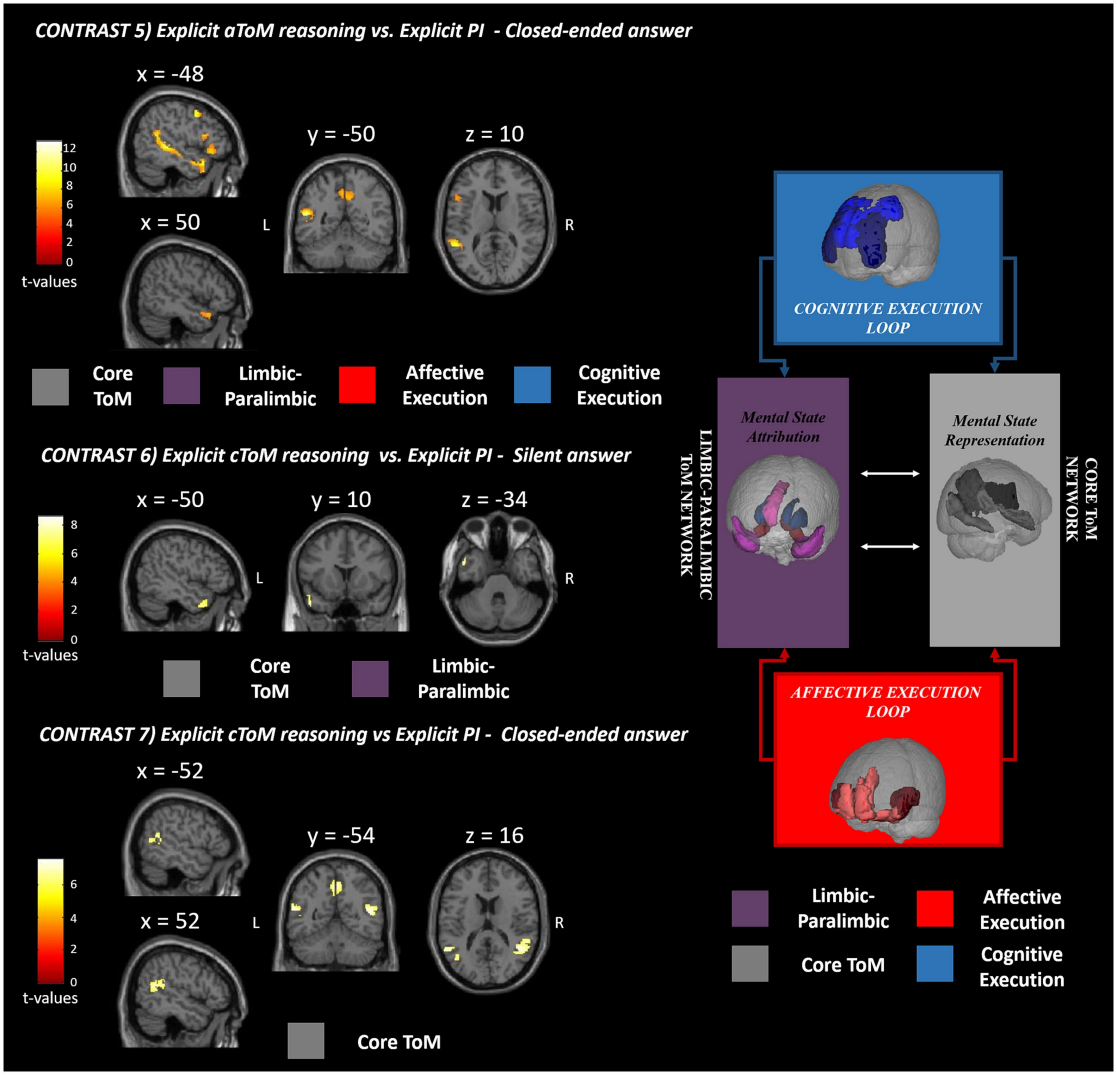
fMRI GLM results. This figure shows the fMRI results observed for silent and closed-ended answering separately for affective (contrast 5) and cognitive (contrasts 6 and 7) ToM reasoning with respect to the physical inference control condition. The significant clusters of activation are reported in red-yellow expressing the *t*-values according to the reported color bar. ToM, theory of mind; PI, physical inference; L, left; R, right. The significant clusters for each contrast are mapped according to the Abu-Akel and Shamay-Tsoory ToM model depicted on the right [adapted from Isernia et al. (2022b)].

The fourth contrast (*explicit aToM reasoning* vs. *explicit silent answer*) did not yield any significant supra-threshold activation, while the fifth contrast (*explicit cToM reasoning* vs. *explicit PI silent answer*) revealed significant functional activations in the bilateral TP (BA 38), precuneus (BA 7), right superior and middle temporal gyri (BA 21), the left inferior frontal cortex (BA 47), specifically the orbital and triangular pars (Figure 4).

As for the sixth contrast (*explicit cToM reasoning* vs. *explicit PI silent answer*), the activations were confined to the left superior and middle TP (BA 21, 38) (Figure 4), while for the seventh contrast (*explicit cToM reasoning* vs. *explicit PI closed-ended answer*), activation was elicited in the bilateral precuneus (BA 7), bilateral superior temporal gyrus (BA 39), and left middle temporal gyrus (BA 39) (Figure 4). As for the contrasts used to directly test for differences between the affective and cognitive ToM components, contrast 8 (*explicit aToM reasoning* vs. *explicit cToM reasoning silent answer*) yielded no significant supra-threshold activation, while contrast 9 (*explicit cToM reasoning* vs. *explicit aToM reasoning silent answer*) showed neural activation located in the bilateral TPJ (BA 39); contrast 10, namely, *explicit aToM reasoning* vs. *explicit cToM reasoning closed-ended answer*, showed significant neural activation in the left superior temporal sulcus, while contrast 11 (explicit cToM reasoning vs. *explicit aToM reasoning closed-ended answer*) revealed no supra-threshold clusters. Detailed results and figures are reported in Supplementary Figure S1.

The ROI-based individual variability analysis revealed low across-subjects consistency with respect to the neural activation elicited by the contrast testing for implicit ToM reasoning. Higher across-subjects consistency was instead observed for the other contrasts specifically in the left TP, left STS, left dorsolateral PFC, bilateral TPJ, and precunei.

The overlap maps and relative histograms are reported in Supplementary Figures S2–S8.

## 4 Discussion

We aimed to explore the brain mechanisms involved in ToM reasoning during everyday social interactions. We adapted the Italian version of the ESCoT into an fMRI task to understand the engagement of ToM neural networks in healthy adults. We hypothesized finding neural activations in the core ToM network and the limbic–paralimbic network. In addition, we predicted finding a neural response in ToM ancillary circuits involving executive frontal loops.

Based on the ToM circuits depicted in the study by Abu-Akel and Shamay-Tsoory (2011), we found no significant neural activation during implicit reasoning on the ESCoT social interaction animations. The lack of activation, which is in contrast with previous studies (Wolf et al., 2010; Jacoby et al., 2016), may be related to the generic social instruction (“*focus on the social interactions”*) provided to participants before viewing the movie. In fact, previous studies (Wolf et al., 2010; Jacoby et al., 2016) used a specific open-ended ToM question as their instruction (“*What is the character thinking?”*). It could be argued that the generic instruction we provided (“*focus on the social interactions*”) may not necessarily direct a participant’s attention to making inferences about mental states, but also different social cues, producing a consequent general social cognition neural pattern in both conditions (ToM and PI). Reversely, the generic instruction prompting physical inference “*focus on elements in the scene*” might not preclude ToM reasoning, and participants could have been similarly engaged in social cognition operations in both ToM and control conditions.

The results of the explicit ToM reasoning demonstrated different activation patterns related to the two response modalities: the open- (silent) and closed-ended answers. The neural activations related to ToM silent answers (contrast 2) were exclusively confined to the left hemisphere and captured significant activations located in the TP and STS (BA 21, 22, 38), suggesting the involvement of the core ToM and limbic–paralimbic networks, devoted to mental state representation and attribution (Abu-Akel and Shamay-Tsoory, 2011). During the ToM closed-ended answering (contrast 3), the neural pattern extended bilaterally in the core and limbic–paralimbic ToM networks (Abu-Akel and Shamay-Tsoory, 2011) and involved additional brain areas of the cognitive and affective execution loops. This brain pattern resembles and extends the one reported by Wolf et al. (2010) during naturalistic social movie tasks in fMRI. The wider neural pattern of closed-ended answers may be linked to the higher specificity of these items compared to the silent questions. In fact, in contrast with closed-ended ones, the silent questions do not prompt reference to a targeted mental state, with consequent interindividual differences in attentional focus and brain activation. In fact, by inviting the participants to silently answer, the spontaneous fluctuation of attention and brain activity (i.e., mind wandering, Braboszcz and Delorme, 2011) may be barely controlled and potentially explains the broader activation of closed-ended than silent answers in eliciting ToM neural patterns. The observed interindividual differences could also be explained by a broader search elicited by an open-ended question versus a closed-ended question, thus resulting in a more varied range of possible responses. The reduced specificity of the silent answer could explain the lack of activations in ToM networks observed during affective ToM answering (contrast 4) and the restricted extension of the neural pattern during cognitive ToM reasoning (contrast 6). Extended activation during multiple-choice answers might also reflect a major recruitment of cognitive processes than silent answers. As a previous fMRI study on mentalizing suggested (Wolf et al., 2010), these answer modes require additional operations, such as interpreting the alternatives, selecting the correct answers, and extensive reading comprehension. These elements concur with higher cognitive demands and task difficulty, plausibly strengthening the brain response. We may assume that to study selective neural patterns within the ToM networks, multiple-choice answers, assuring a more effective neural response, are preferable.

The lack of activation during affective ToM (contrast 4), as opposed to a restricted minimal pattern during cognitive ToM silent answers, might be ascribed, according to our supplementary analyses, to higher inter-subject variability in affective ToM reasoning resulting in a more widespread and less consistent neural pattern component (see Supplementary Figures S5, S7). These distinct patterns may be partially explained by the different types of mental states on which the subject is invited to reflect emotions versus thoughts. In fact, a previous study (Drobyshevsky et al., 2006) exploring the sensitivity of common fMRI tasks assessing different neurocognitive domains reported poor sensitivity for the task on emotional function, which did not monitor the subject’s performance.

We found that closed answering enabled distinct neural patterns related to affective and cognitive mental state reasoning. The neural activation of affective ToM (contrast 5) included bilaterally the core and limbic–paralimbic networks, including the precunei, anterior superior temporal sulci, and TPs (BA 7, 21, 38). The involvement of the core network extended to the posterior portion of the STS only in the left hemisphere. The significant cluster of activation included some regions of the affective and cognitive execution loops: the left inferior frontal gyrus (pars orbitalis and triangularis, BA 47), the insula, and the middle frontal gyrus. This pattern of activation resembles the brain network involved in affective mental state representation, attribution, and application (Abu-Akel and Shamay-Tsoory, 2011). Interestingly, the neural activation cluster located in the insula confirms the specific involvement of emotional content and social behavior processing during the execution of an affective naturalistic ToM task (Henry et al., 2016; de Oliveira-Souza and Moll, 2019).

Concerning the cognitive mental state neural patterns (contrast 7), the closed-ended answers yielded brain activations located only in the core ToM network, including the bilateral TPJ and precunei (BA 39, 7). Again, the ToM core network for mental state representation was effectively elicited as expected, but the limbic–paralimbic circuit and the frontal loop supporting cognitive mental state deployment were not included. The lack of involvement of the frontal brain areas may be related to several aspects. First, these areas may be recruited both in physical inference and ToM answering using a complex ecological stimulus. The absence of the cognitive frontal circuit could be ascribed to the preserved cognitive level of participants (healthy adults) included in the study, plausibly showing a high level of frontal executive functioning. Preserved and high social cognition processes may lead to selective involvement of specific ToM circuits but not the executive control ones. In addition, it is worth noting that the task itself requires reasoning on the mental states of social events that have already occurred, and, unlike other tests, it does not involve inferring or anticipating future intentions and behaviors of the characters, which is likely to engage the frontal loop. A recent meta-analysis (Schurz et al., 2021) presented distinct patterns of neural activation based on social cognition test stimuli, proposing a three clustering solution: cognitive (ToM), affective (empathy), and intermediate domains. According to the present results, the ESCoT belongs to the intermediate cluster, bridging between the different domains of cognitive ToM and empathy. The intermediate positioning of ESCoT may be linked to its intrinsically ecological nature. In fact, by showing everyday life interactions, cognitive and affective ToM reasoning might be at least in part simultaneously elicited to properly interpret the social dynamics.

Finally, regarding the core ToM network activation during affective and cognitive ToM answering, a distinct neural pattern was also observed in the temporal areas. Especially the STS activation (BA 21/22) was found only during the affective ToM answering, while the TPJ involvement (BA 39) was observed exclusively in the cognitive ToM reasoning. These findings are further confirmed by explorinsg a direct comparison between the cognitive and affective ToM neural patterns (contrasts 8–11). In fact, affective ToM revealed neural activation located in the STS when explicit ToM reasoning was explored using closed-ended answering, while cognitive ToM reasoning relied on the activation of the bilateral TPJ when explicit ToM reasoning was tested using silent answering.

The TPJ has been widely reported as a crucial hub for the switching between self-perspective and others’ viewpoint during mentalizing tasks (Decety and Sommerville, 2003; Uddin et al., 2005; Corbetta et al., 2008), playing a mediating role between self and others’ perspectives (Schulte-Rüther et al., 2007). With the ESCoT scenarios, participants are invited to reflect on the mental states of characters who interact in a specific context, which is influenced by social norm adhesion or violation. When participants are prompted to reason on the affective mental states of the injured character in the scenario (e.g., the old woman who does not receive help from the young man), attention was easily directed to that character with whom the participant was prone to empathize, resulting in neural activation confined in the STS. Instead, when the participant is invited to reason on the thoughts and expectations of the injured character (i.e., in the previous example, did the young man behave as the old woman expected?), the level of complexity increases because it requires the switching to others’ viewpoints. In fact, the participant likely compares the characters’ violated expectations, in line with their own, with the intentions of the other character who violated those expectations. In this case, we observed major involvement of TPJ, as previously reported.

Another explanation might be related to previous studies supporting that the ToM circuit is prone to respond more to unpredicted (violation) than predicted (adhesion) information (Cloutier et al., 2011; Dungan et al., 2016), in light of the predictive coding view of the social brain. In addition, evidence highlighted an enhanced activity of the bilateral TPJ when mental state information is relevant for moral judgment (Young and Waytz, 2013). Especially, enhanced activations in the TPJ are observed when an immoral behavior is shown (Kim et al., 2021) compared to a moral social comportment. A suggested explanation is the perception of immoral behavior as containing more intents than moral ones (Brambilla et al., 2019), plausibly recruiting a broader neural circuit. Overall, the role of ToM in moral cognition has been reported in the literature (Turiel, 1983). In fact, reasoning on people’s intentionality inflects the moral judgment when the individual is wondering whether to blame an agent’s behavior or not (Knobe, 2005).

The adaptation of the ESCoT into an fMRI task was not without limitations which should be addressed in future studies, especially involving aging or clinical populations. First, the ToM silent answering resulted in poor neural activation as previously demonstrated (Wolf et al., 2010). Therefore, only closed-ended questions should be included in future versions. The modification will also shorten the overall duration of the experiment. In addition, the high socio-educational level of participants may have partly prevented the generalization of the observed results. Moreover, the differentiation of the neural underpinnings related to the adhesion or violation of the social norms depicted in the social interaction animations was not investigated in this study, which may potentially affect ToM mechanisms. Future contributions should address these aspects to explore and deepen the knowledge of the neural correlates involved in social cognition deficits. Future studies could use this version of the ESCoT in clinical settings to study ToM network in different pathologies at risk or with social cognition deficits. This could include neurodegenerative conditions, schizophrenia, or developmental disorders. In addition, the task could be used to evaluate neuroplasticity or reorganization after ToM training such as those used in rehabilitative settings. Finally, one last aspect that should not be overlooked when exploring higher-level cognitive domains such as ToM reasoning is the issue of individual variability (Saxe and Kanwisher, 2003; Saxe and Powell, 2006). To address this issue, standard GLM factorial analysis should be integrated with further analysis assessing the consistency of brain responses across subjects. This has been addressed in the present study by performing an additional analysis with the methods proposed in a study by Seghier and Price (2016), and the findings corroborate the group-level results. Specifically, concerning the two ToM components, the differential involvement of TPJ in cToM and STS in aToM was substantiated.

Finally, it is worth mentioning that our results prevented us from investigating online mentalizing reasoning when the subject is involved in first person social interactions. Future contributions might consider adopting different acquisition settings such as hyper-scanning (e.g., Bazán and Amaro, 2022 for a review) to study neural patterns during social interaction.

## 5 Conclusion

In conclusion, the present study investigated the brain mechanisms involved in individual mentalizing reasoning in real-life social interactions. The ESCoT stimuli were particularly effective in evoking ToM core neural underpinnings and elicited executive frontal loops. These results support the application of the ESCoT as a sensitive test of social cognition and provide further insights into the neural regions involved in social cognition.

## Data availability statement

The raw data supporting the conclusions of this article will be made available by the authors, without undue reservation.

## Ethics statement

The studies involving humans were approved by Fondazione Don Gnocchi-Milan Ethics Committee. The studies were conducted in accordance with the local legislation and institutional requirements. The participants provided their written informed consent to participate in this study. Written informed consent was obtained from the individual(s) for the publication of any potentially identifiable images or data included in this article.

## Author contributions

SI: Conceptualization, Formal analysis, Methodology, Writing – original draft, Writing – review & editing. AP: Formal analysis, Methodology, Writing – original draft, Writing – review & editing. FR: Methodology, Writing – review & editing. DC: Formal analysis, Writing – review & editing. MC: Data curation, Writing – review & editing. VB: Methodology, Supervision, Writing – review & editing. RB: Writing – review & editing. SM: Writing – review & editing. FB: Funding acquisition, Supervision, Writing – review & editing.

## Footnotes

^1^https://pstnet.com/products/e-prime/

^2^https://www.nordicneurolab.com/

^3^https://www.fil.ion.ucl.ac.uk/spm/

^4^https://it.mathworks.com/products/matlab.html

^5^https://fsl.fmrib.ox.ac.uk/fsl/fslwiki/
