# Supplementary material for: Human reasoning on social interactions in ecological contexts: insights from the theory of mind brain circuits
Source: Front Neurosci. 2024 Aug 1;18:1420122. doi: 10.3389/fnins.2024.1420122 (PMC11339883; doi:10.3389/fnins.2024.1420122)
Supplement: Supplementary file 1 [file Data_Sheet_1.docx]

**Human reasoning on social interactions in ecological contexts: insights from the theory of mind brain circuits**

**Supplementary Material**

**S1. Results of the direct comparison of cognitive and affective ToM components**

Four further contrasts have been computed directly comparing the cognitive and affective components of ToM reasoning, namely: *8) explicit aToM reasoning vs explicit cToM reasoning – silent answer, 9) explicit cToM reasoning vs explicit aToM reasoning – silent answer, 10) explicit aToM reasoning vs explicit cToM reasoning – closed-ended answer, 11) explicit cToM reasoning vs explicit aToM reasoning – closed-ended answer*.

The activation clusters are reported in Figure S1 for the threshold pFWE<0.05, cluster size = 30 voxels.

Specifically, the following activations were retrieved:

*8) explicit aToM reasoning vs explicit cToM reasoning – silent answer- pFWE<0.05: NO SUPRA-THRESHOLD CLUSTERS.*

*9) explicit cToM reasoning vs explicit aToM reasoning – silent answer - pFWE<0.05: bilateral temporo-parietal junction.*

*10) explicit aToM reasoning vs explicit cToM reasoning – closed-ended answer - pFWE<0.05: left superior temporal sulcus.*

*11) explicit cToM reasoning vs explicit aToM reasoning – closed-ended answer* *- pFWE<0.05: NO SUPRA-THRESHOLD CLUSTERS.*

**
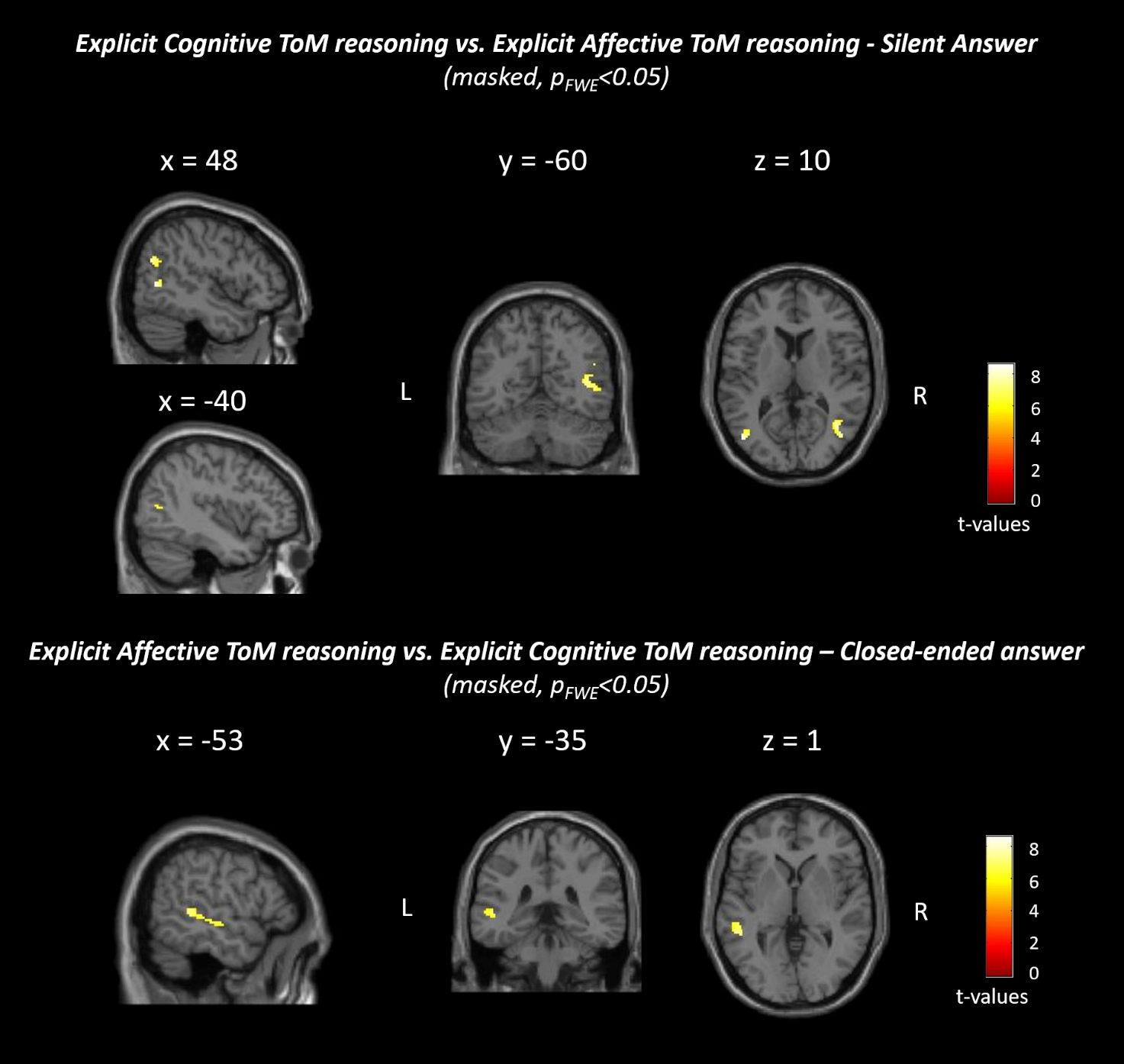
**

**Figure S1**. Direct comparison explicit ToM reasoning – (Top panel) Silent answer: Cognitive *ToM reasoning vs. Affective ToM reasoning;* (Bottom Panel) Direct comparison Closed-ended answer: *Affective ToM reasoning vs. Cognitive To*M *reasoning*.

**S2. Individual variability methods and results**

To complement the classical GLM factorial analysis, subjects’ inter-individual variability was performed. Specifically, subject-specific activation maps derived for all the above-mentioned contrasts have been used to compute threshold-dependent overlap maps representing the proportion of subject activation in a given region of interest (ROI) (Seghier et al., 2016). The ROIs were defined according to an in-house developed atlas-derived inclusive mask previously described in (Isernia, Pirastru, et al., 2022), comprising the cerebral areas relevant to ToM reasoning according to Abu-Akel and Shamay-Tsoory's model (Abu-Akel & Shamay-Tsoory, 2011). The subject specific activation maps were derived according to the following thresholds: p_unc_ < 0.001 cluster-size ≥ 30.

The ROI-based individual variability analysis revealed low consistency with respect to the neural activation elicited by contrast 1 testing the implicit ToM reasoning (i.e., *implicit ToM reasoning vs implicit PI*). Intermediate consistency was instead observed for the explicit ToM reasoning elicited by the silent answering contrast (i.e., *2 explicit ToM reasoning vs explicit PI silent answer*), with a higher percentage of subjects showing neural activation in the left temporal pole. The highest consistency was relative to the contrast investigating the explicit ToM reasoning exploiting closed-ended answers (i.e., *contrast 3*), which showed peaks of higher percentages of subjects showing neural activation in the left temporo-parietal junction.

As to the contrasts separately investigating the affective and cognitive ToM dimension: highest consistency was found in the left STS for contrast 4 (*i.e. explicit aToM reasoning vs explicit PI silent answer*) and contrast 5 (i.e., *explicit aToM reasoning vs explicit PI closed-ended answer*), the latter also revealed higher consistency for left dorsolateral PFC; while we found highest consistency in the left TP and bilateral TPJ and bilateral precuneus and TPJ for contrast 6 (*i.e., explicit cToM reasoning vs explicit PI silent answer*) and 7 (i.e., *explicit cToM reasoning vs explicit PI closed-ended answer*) respectively.

These results are in line with group-level statistical maps.

The overlap maps and relative histograms are reported in the supplementary materials (Figures S2 to S8).


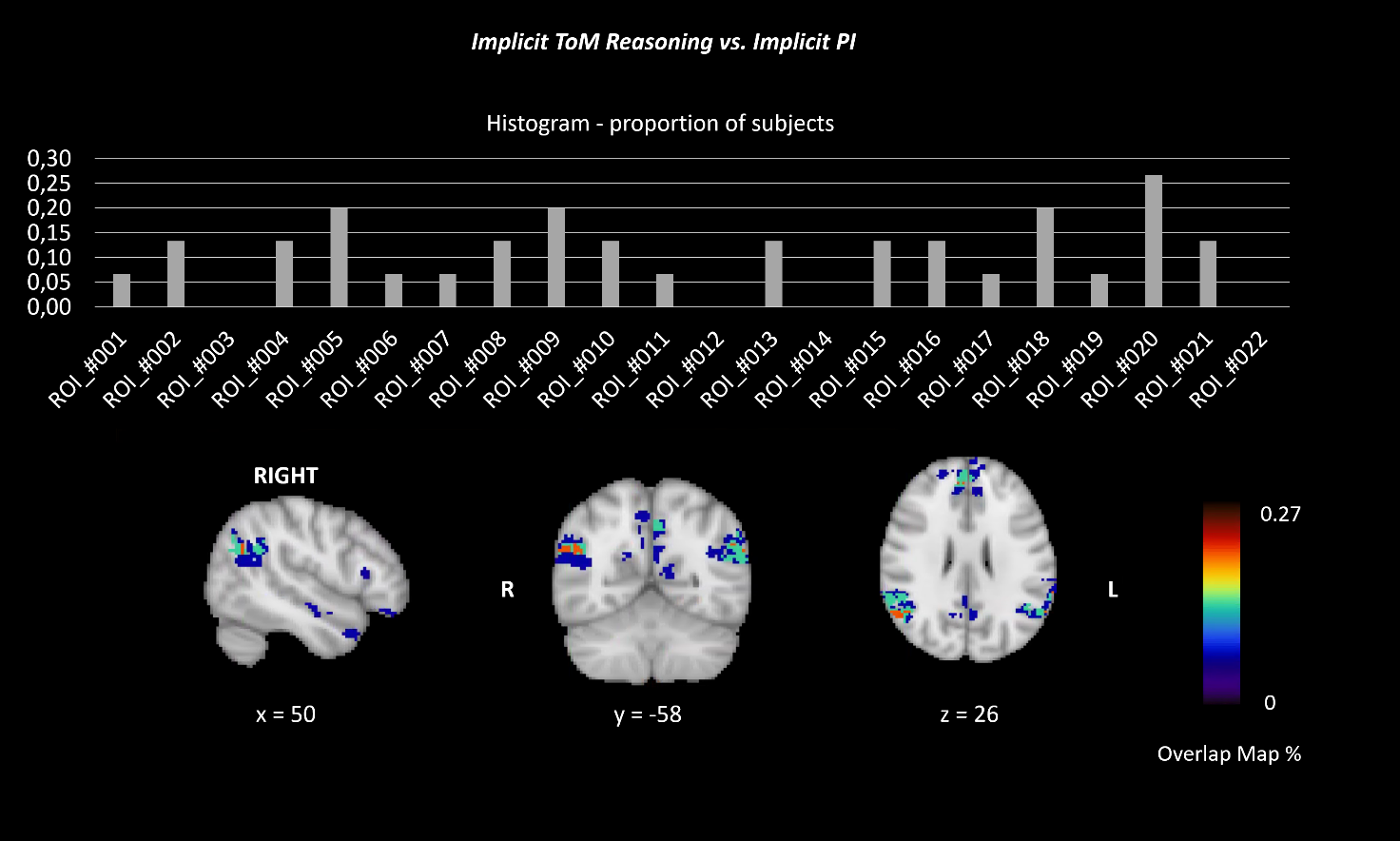


**Figure S2.** Histogram (top panel) and overlap maps (bottom panel) measuring consistency derived from subject-specific activation maps (punc<0.001, k=30, restricted to ToM neural model mask as depicted in Isernia, Pirastru, et al., 2022) for the contrast 1_ implicit ToM reasoning vs implicit PI.

Legend: ROI_#001 = left anterior cingulate cortex; ROI_#002 = left anterior part of the superior temporal sulcus; ROI_#003 = left dorsal striatum; ROI_#004 = left dorso-lateral prefrontal cortex; ROI_#005 = left dorso-medial prefrontal cortex; ROI_#006 = left infero-lateral prefrontal cortex; ROI_#007 = left orbitofrontal and ventromedial prefrontal cortices; ROI_#008 = left precuneus and posterior cingulate cortex; ROI_#009 = left temporo-parietal junction; ROI_#010 = left temporal pole; ROI_#011 = left ventral striatum and amygdala; ROI_#012 = right anterior cingulate cortex; ROI_#013 = righte anterior part of the superior temporal sulcus; ROI_#014 = right dorsal striatum; ROI_#015 = right dorso-lateral prefrontal cortex; ROI_#016 = right dorso-medial prefrontal cortex; ROI_#017 = right infero-lateral prefrontal cortex; ROI_#018 = right orbitofrontal and ventromedial prefrontal cortices; ROI_#019 = right precuneus and posterior cingulate cortex; ROI_#020 = right temporo-parietal junction; ROI_#021 = right temporal pole; ROI_#022 = right ventral striatum and amygdala.

**
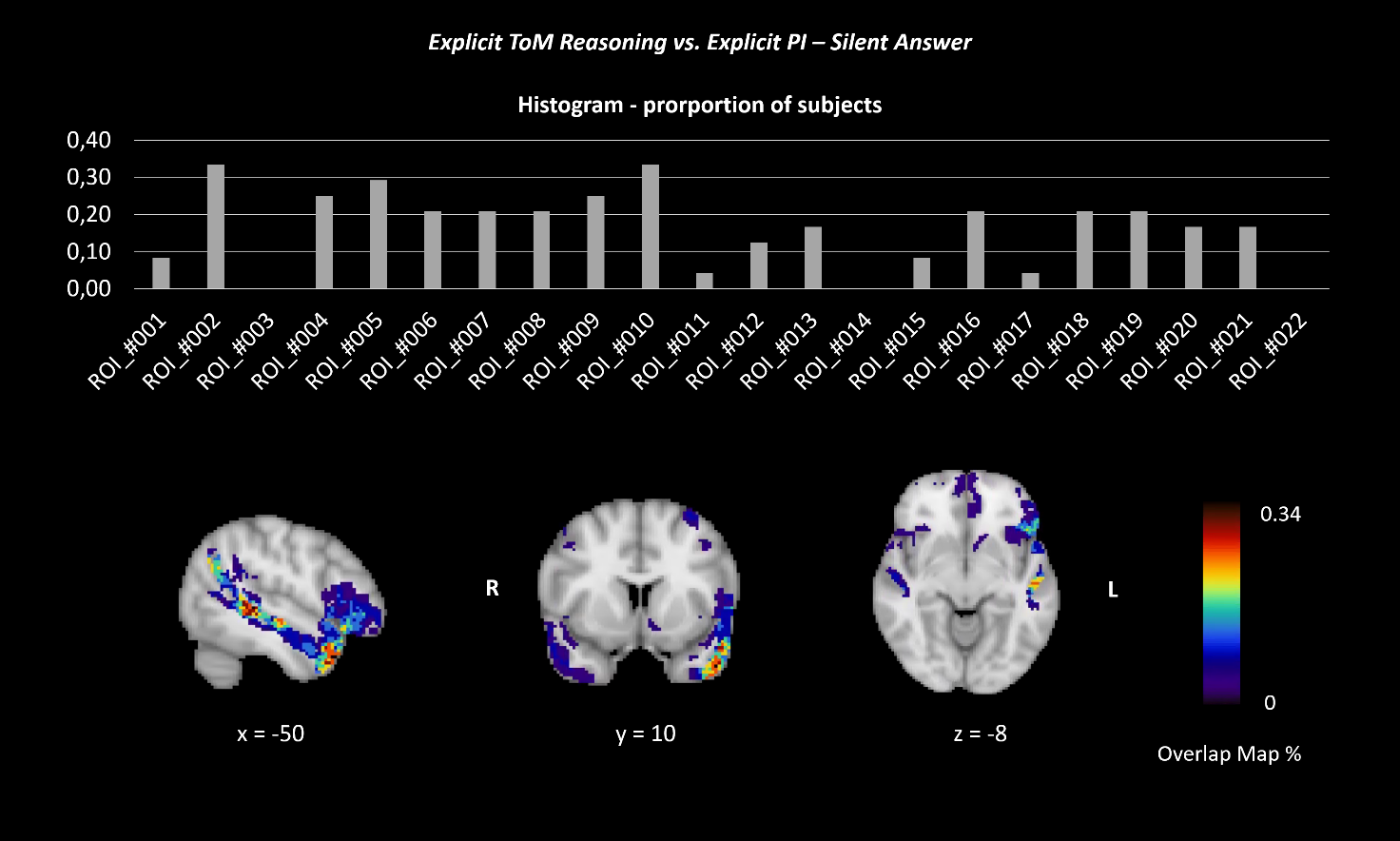
**

**Figure S3.** Histogram (top panel) and overlap maps (bottom panel) measuring consistency derived from subject-specific activation maps (punc<0.001, k=30, restricted to ToM neural model mask as depicted in Isernia, Pirastru, et al., 2022) for the contrast 2_ explicit ToM reasoning vs explicit PI salient answer.

Legend: ROI_#001 = left anterior cingulate cortex; ROI_#002 = left anterior part of the superior temporal sulcus; ROI_#003 = left dorsal striatum; ROI_#004 = left dorso-lateral prefrontal cortex; ROI_#005 = left dorso-medial prefrontal cortex; ROI_#006 = left infero-lateral prefrontal cortex; ROI_#007 = left orbitofrontal and ventromedial prefrontal cortices; ROI_#008 = left precuneus and posterior cingulate cortex; ROI_#009 = left temporo-parietal junction; ROI_#010 = left temporal pole; ROI_#011 = left ventral striatum and amygdala; ROI_#012 = right anterior cingulate cortex; ROI_#013 = righte anterior part of the superior temporal sulcus; ROI_#014 = right dorsal striatum; ROI_#015 = right dorso-lateral prefrontal cortex; ROI_#016 = right dorso-medial prefrontal cortex; ROI_#017 = right infero-lateral prefrontal cortex; ROI_#018 = right orbitofrontal and ventromedial prefrontal cortices; ROI_#019 = right precuneus and posterior cingulate cortex; ROI_#020 = right temporo-parietal junction; ROI_#021 = right temporal pole; ROI_#022 = right ventral striatum and amygdala.


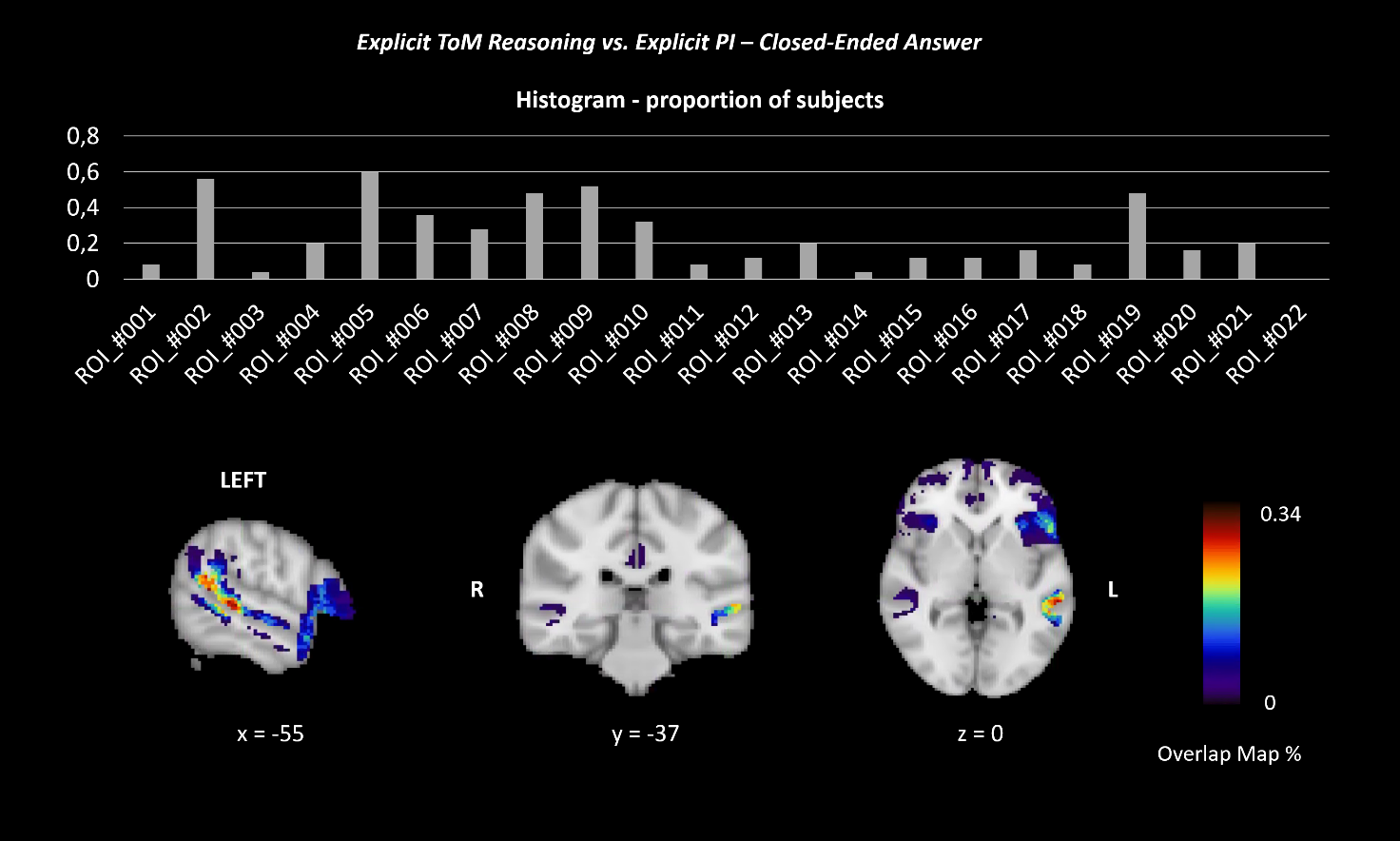


**Figure S4.** Histogram (top panel) and overlap maps (bottom panel) measuring consistency derived from subject-specific activation maps (punc<0.001, k=30, restricted to ToM neural model mask as depicted in Isernia, Pirastru, et al., 2022) for the contrast 3_ explicit ToM reasoning vs explicit PI closed-ended answer.

Legend: ROI_#001 = left anterior cingulate cortex; ROI_#002 = left anterior part of the superior temporal sulcus; ROI_#003 = left dorsal striatum; ROI_#004 = left dorso-lateral prefrontal cortex; ROI_#005 = left dorso-medial prefrontal cortex; ROI_#006 = left infero-lateral prefrontal cortex; ROI_#007 = left orbitofrontal and ventromedial prefrontal cortices; ROI_#008 = left precuneus and posterior cingulate cortex; ROI_#009 = left temporo-parietal junction; ROI_#010 = left temporal pole; ROI_#011 = left ventral striatum and amygdala; ROI_#012 = right anterior cingulate cortex; ROI_#013 = righte anterior part of the superior temporal sulcus; ROI_#014 = right dorsal striatum; ROI_#015 = right dorso-lateral prefrontal cortex; ROI_#016 = right dorso-medial prefrontal cortex; ROI_#017 = right infero-lateral prefrontal cortex; ROI_#018 = right orbitofrontal and ventromedial prefrontal cortices; ROI_#019 = right precuneus and posterior cingulate cortex; ROI_#020 = right temporo-parietal junction; ROI_#021 = right temporal pole; ROI_#022 = right ventral striatum and amygdala.


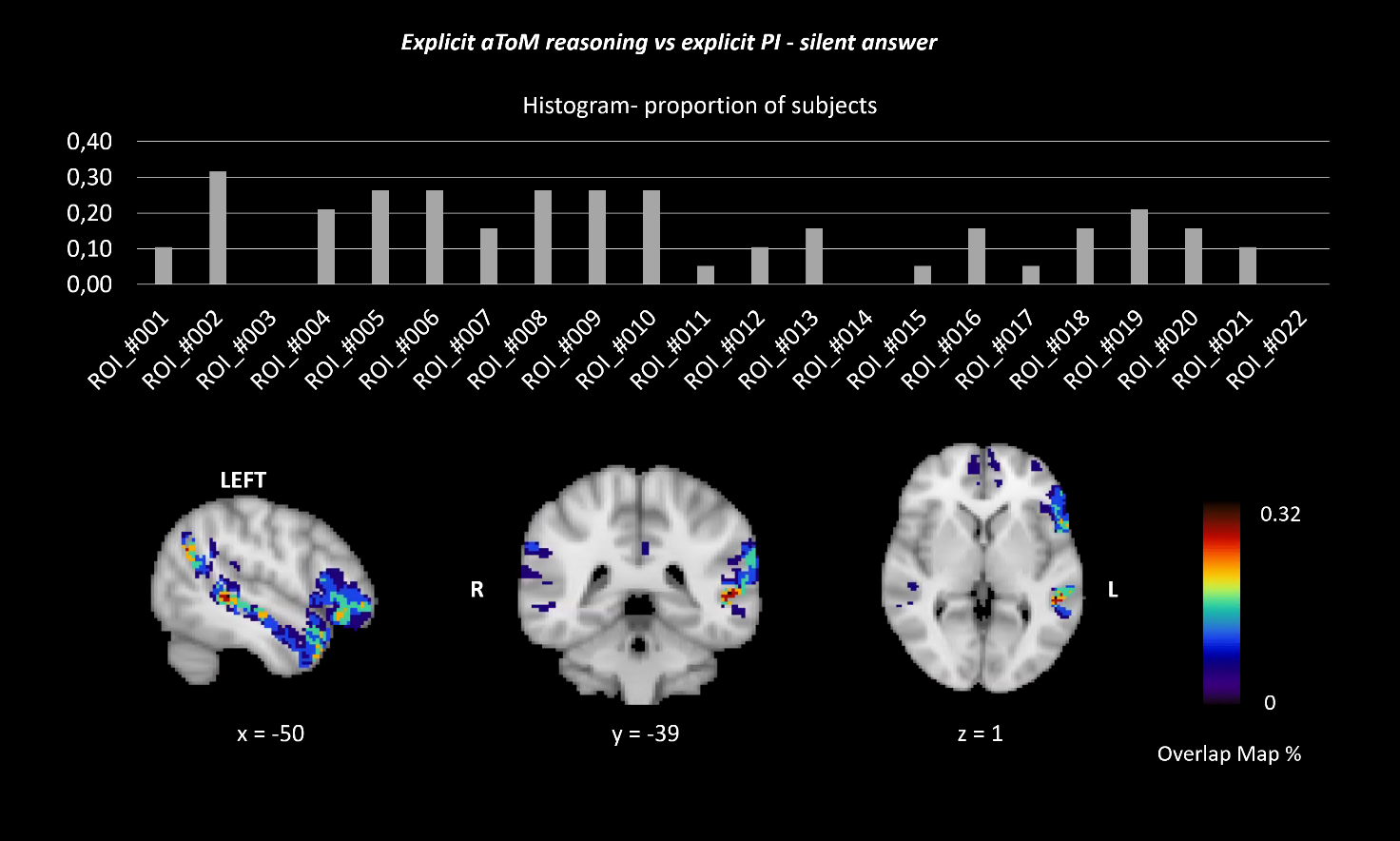


**Figure S5**. Histogram (top panel) and overlap maps (bottom panel) measuring consistency derived from subject-specific activation maps (punc<0.001, k=30, restricted to ToM neural model mask as depicted in Isernia, Pirastru, et al., 2022) for the contrast 4) explicit aToM reasoning vs explicit PI silent answer.

Legend: ROI_#001 = left anterior cingulate cortex; ROI_#002 = left anterior part of the superior temporal sulcus; ROI_#003 = left dorsal striatum; ROI_#004 = left dorso-lateral prefrontal cortex; ROI_#005 = left dorso-medial prefrontal cortex; ROI_#006 = left infero-lateral prefrontal cortex; ROI_#007 = left orbitofrontal and ventromedial prefrontal cortices; ROI_#008 = left precuneus and posterior cingulate cortex; ROI_#009 = left temporo-parietal junction; ROI_#010 = left temporal pole; ROI_#011 = left ventral striatum and amygdala; ROI_#012 = right anterior cingulate cortex; ROI_#013 = righte anterior part of the superior temporal sulcus; ROI_#014 = right dorsal striatum; ROI_#015 = right dorso-lateral prefrontal cortex; ROI_#016 = right dorso-medial prefrontal cortex; ROI_#017 = right infero-lateral prefrontal cortex; ROI_#018 = right orbitofrontal and ventromedial prefrontal cortices; ROI_#019 = right precuneus and posterior cingulate cortex; ROI_#020 = right temporo-parietal junction; ROI_#021 = right temporal pole; ROI_#022 = right ventral striatum and amygdala.


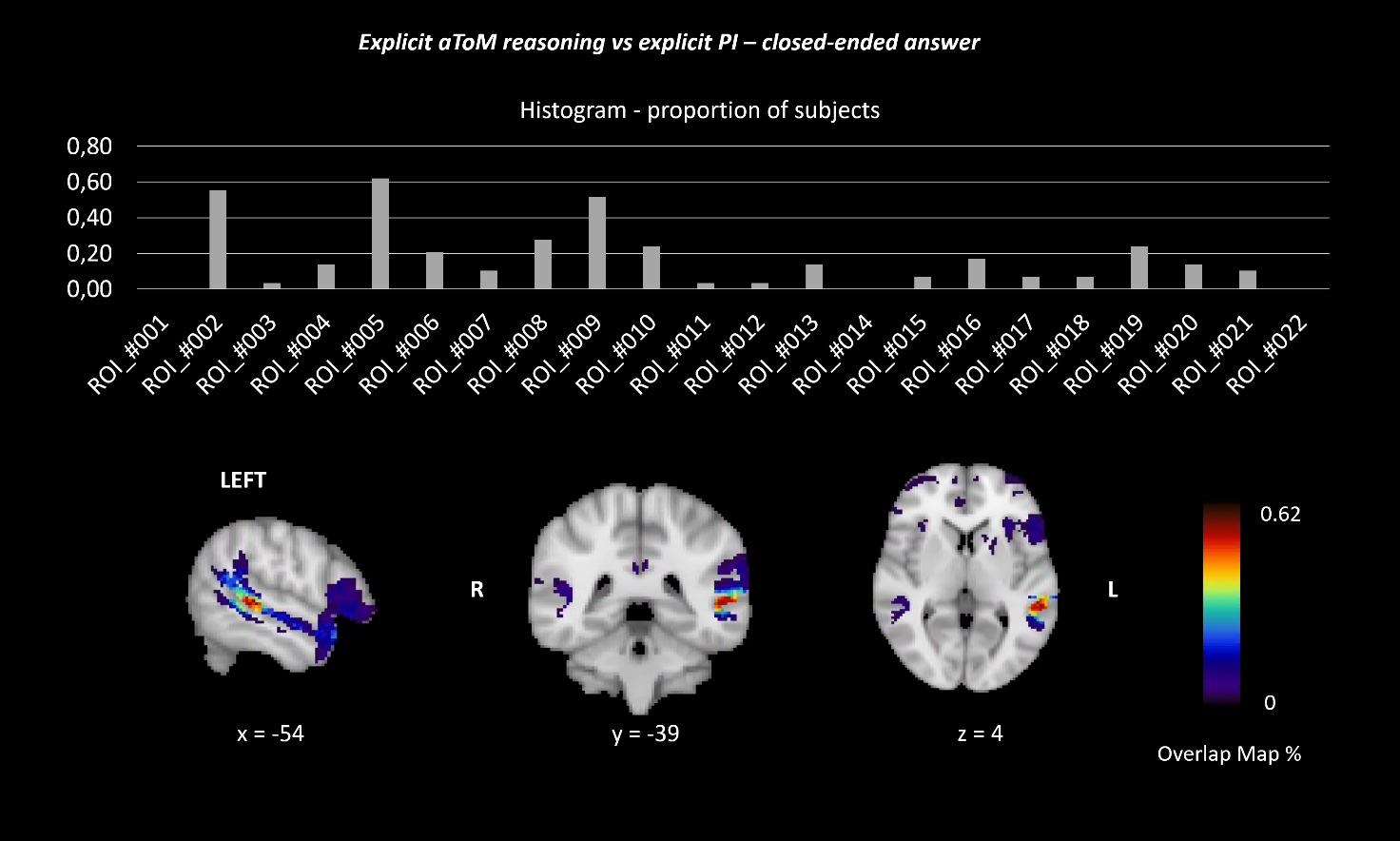


**Figure S6**. Histogram (top panel) and overlap maps (bottom panel) measuring consistency derived from subject-specific activation maps (punc<0.001, k=30, restricted to ToM neural model mask as depicted in Isernia, Pirastru, et al., 2022) for the contrast 5) explicit aToM reasoning vs explicit PI closed-ended answer.

Legend: ROI_#001 = left anterior cingulate cortex; ROI_#002 = left anterior part of the superior temporal sulcus; ROI_#003 = left dorsal striatum; ROI_#004 = left dorso-lateral prefrontal cortex; ROI_#005 = left dorso-medial prefrontal cortex; ROI_#006 = left infero-lateral prefrontal cortex; ROI_#007 = left orbitofrontal and ventromedial prefrontal cortices; ROI_#008 = left precuneus and posterior cingulate cortex; ROI_#009 = left temporo-parietal junction; ROI_#010 = left temporal pole; ROI_#011 = left ventral striatum and amygdala; ROI_#012 = right anterior cingulate cortex; ROI_#013 = righte anterior part of the superior temporal sulcus; ROI_#014 = right dorsal striatum; ROI_#015 = right dorso-lateral prefrontal cortex; ROI_#016 = right dorso-medial prefrontal cortex; ROI_#017 = right infero-lateral prefrontal cortex; ROI_#018 = right orbitofrontal and ventromedial prefrontal cortices; ROI_#019 = right precuneus and posterior cingulate cortex; ROI_#020 = right temporo-parietal junction; ROI_#021 = right temporal pole; ROI_#022 = right ventral striatum and amygdala.


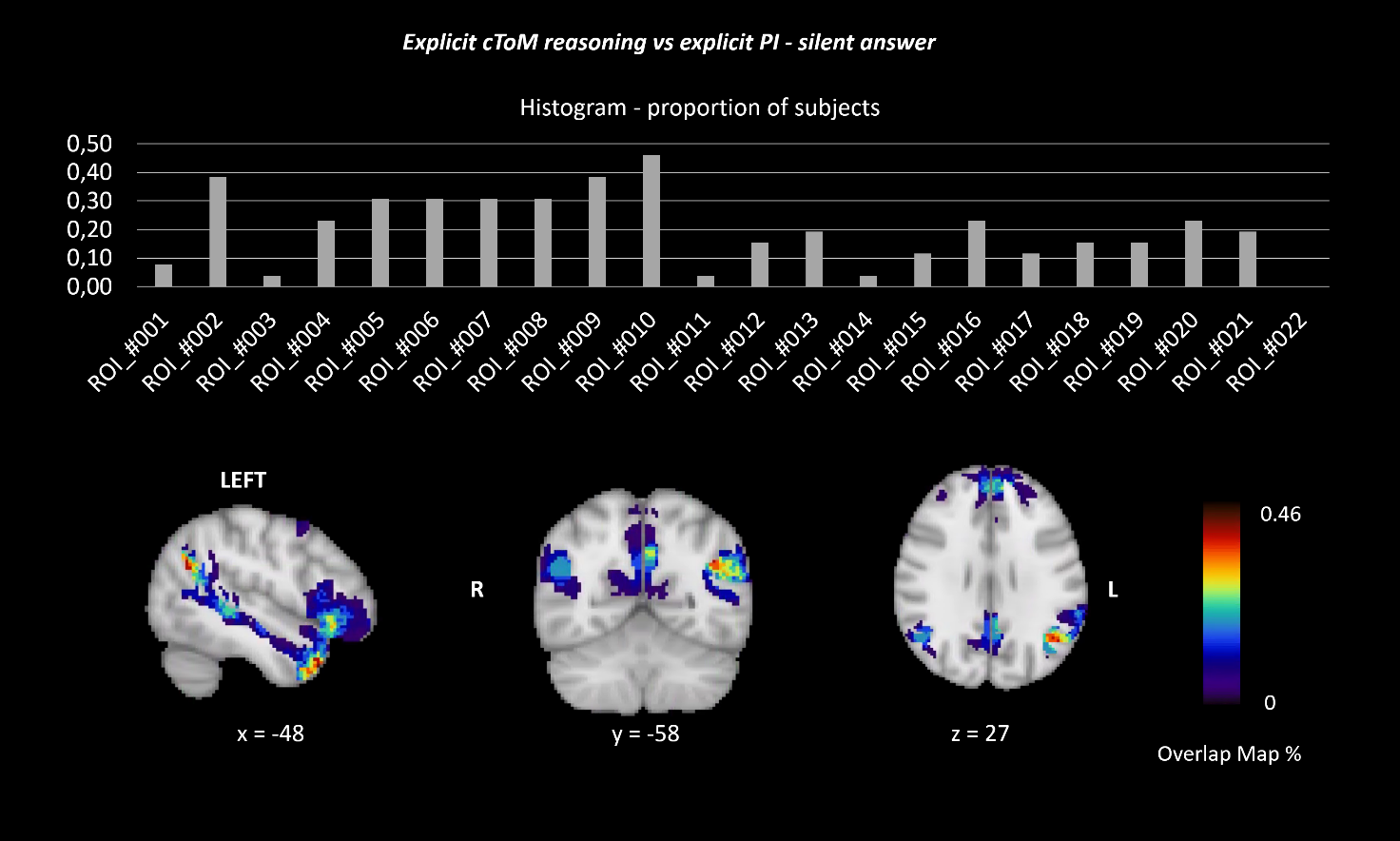


**Figure S7**. Histogram (top panel) and overlap maps (bottom panel) measuring consistency derived from subject-specific activation maps (punc<0.001, k=30, restricted to ToM neural model mask as depicted in Isernia, Pirastru, et al., 2022) for the contrast 6) explicit cToM reasoning vs explicit PI silent answer.

Legend: ROI_#001 = left anterior cingulate cortex; ROI_#002 = left anterior part of the superior temporal sulcus; ROI_#003 = left dorsal striatum; ROI_#004 = left dorso-lateral prefrontal cortex; ROI_#005 = left dorso-medial prefrontal cortex; ROI_#006 = left infero-lateral prefrontal cortex; ROI_#007 = left orbitofrontal and ventromedial prefrontal cortices; ROI_#008 = left precuneus and posterior cingulate cortex; ROI_#009 = left temporo-parietal junction; ROI_#010 = left temporal pole; ROI_#011 = left ventral striatum and amygdala; ROI_#012 = right anterior cingulate cortex; ROI_#013 = righte anterior part of the superior temporal sulcus; ROI_#014 = right dorsal striatum; ROI_#015 = right dorso-lateral prefrontal cortex; ROI_#016 = right dorso-medial prefrontal cortex; ROI_#017 = right infero-lateral prefrontal cortex; ROI_#018 = right orbitofrontal and ventromedial prefrontal cortices; ROI_#019 = right precuneus and posterior cingulate cortex; ROI_#020 = right temporo-parietal junction; ROI_#021 = right temporal pole; ROI_#022 = right ventral striatum and amygdala.


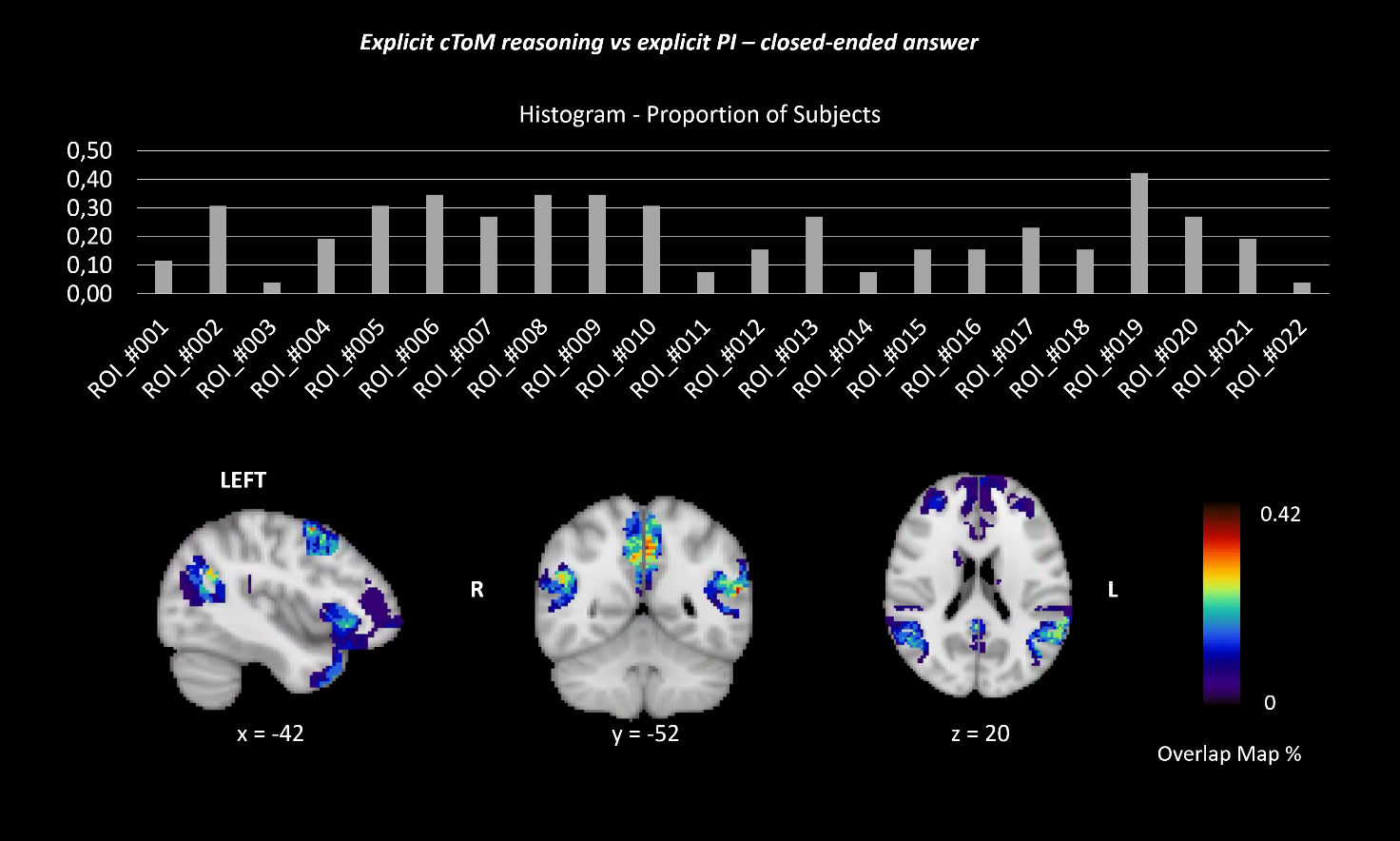


**Figure S8**. Histogram (top panel) and overlap maps (bottom panel) measuring consistency derived from subject-specific activation maps (punc<0.001, k=30, restricted to ToM neural model mask as depicted in Isernia, Pirastru, et al., 2022) for the contrast 7) explicit cToM reasoning vs explicit PI closed-ended answer.

Legend: ROI_#001 = left anterior cingulate cortex; ROI_#002 = left anterior part of the superior temporal sulcus; ROI_#003 = left dorsal striatum; ROI_#004 = left dorso-lateral prefrontal cortex; ROI_#005 = left dorso-medial prefrontal cortex; ROI_#006 = left infero-lateral prefrontal cortex; ROI_#007 = left orbitofrontal and ventromedial prefrontal cortices; ROI_#008 = left precuneus and posterior cingulate cortex; ROI_#009 = left temporo-parietal junction; ROI_#010 = left temporal pole; ROI_#011 = left ventral striatum and amygdala; ROI_#012 = right anterior cingulate cortex; ROI_#013 = righte anterior part of the superior temporal sulcus; ROI_#014 = right dorsal striatum; ROI_#015 = right dorso-lateral prefrontal cortex; ROI_#016 = right dorso-medial prefrontal cortex; ROI_#017 = right infero-lateral prefrontal cortex; ROI_#018 = right orbitofrontal and ventromedial prefrontal cortices; ROI_#019 = right precuneus and posterior cingulate cortex; ROI_#020 = right temporo-parietal junction; ROI_#021 = right temporal pole; ROI_#022 = right ventral striatum and amygdala.
